# Genetic analysis of the NifM dependence of the nitrogenase iron proteins

**DOI:** 10.1128/mbio.02642-25

**Published:** 2025-10-31

**Authors:** Zhuoting Xie, Shuyi Cai, Haoyang Chen, Shuyuan Kong, Letian Tang, Yi-Ping Wang, Jianguo Yang

**Affiliations:** 1 State Key Laboratory of Gene Function and Modulation Research, School of Advanced Agricultural Sciences, Peking University 12465, Beijing, China; 2 College of Plant Protection, China Agricultural University 539443 , Beijing, China; 3 College of Biological Sciences and Technology, Beijing Forestry University 12380, Beijing, China; 4 Yazhouwan National Laboratory 735983 , Sanya, China; University of California Berkeley, Berkeley, California, USA

**Keywords:** nitrogenase, iron protein, NifM

## Abstract

NifH maturation in certain diazotrophic bacteria depends on NifM, a protein with a PpiC-isomerase domain. Intriguingly, the putative proline substrate for NifM is present in NifH proteins from strains lacking *nifM*, raising questions regarding the actual role of NifM and the necessity of isomerases for the maturation of NifH proteins in organisms devoid of *nifM*. Here, we established that the C-terminal PpiC-isomerase domain of NifM is predominant in NifH maturation, whereas the N-terminal region may facilitate this process by binding to NifH. Proline scanning indicated that Pro^256^ of NifH is more likely involved in NifM-directed maturation than the previously proposed Pro^258^, as NifH variants from *Klebsiella oxytoca* with P258I, T, or V substitutions retained full activity while still requiring NifM for function. Conversely, alterations of Pro^256^ to any amino acid resulted in loss of function and insolubility. We subsequently assessed the NifM dependence of 15 NifHs from diverse bacteria based on the reconstituted nitrogenase system in *Escherichia coli*. Additionally, we validated the NifM independence of a selected NifH from *Bacillus* in *E. coli* strains deficient in *ppiC*, *ppiD*, and *surA* genes, both individually and in combination, as these gene products also contain a PpiC-isomerase domain. In conclusion, our findings rectified earlier misconceptions about the catalytic site in NifH for NifM and demonstrated that the PpiC isomerase domain is not required for the maturation of the NifH protein in bacteria without *nifM*. Moreover, the NifM-independent NifH, identified in this study, has the potential to engineer diazotrophs in heterogeneous hosts.

**IMPORTANCE:**

The iron protein NifH is one of the most important components of the nitrogenase system, contributing to cofactor biosynthesis and acting as an obligate electron donor for nitrogenase under *in vivo* conditions. Unraveling the maturation mechanism of NifH holds substantial significance for both theoretical and practical research. Our investigation into the dependence of NifM on NifH maturation has yielded novel insights into the function of NifM and has corrected previous misconceptions regarding the catalytic site in NifH for NifM. The prospect of engineering diazotrophs in heterologous hosts, particularly crops, through the direct transfer of the nitrogenase system represents an area of considerable scientific interest. Notably, the isomerase-independent NifH identified in this study may offer advantages in heterologous expression scenarios as it requires fewer accessory proteins for functionality.

## INTRODUCTION

Biological nitrogen fixation plays a critical role in global nitrogen cycling and has significant agricultural implications (1, 2). The ability to fix biological nitrogen depends on the nitrogenase system and is limited to a select group of bacteria and archaea (3). Three different types of nitrogenase systems, molybdenum (MoFe), vanadium (VFe), and iron-only (FeFe), have been identified in nature, depending on the heterometallic composition of the active site cofactor (4). The MoFe nitrogenase system has been studied extensively and is present in all diazotrophic organisms sequenced to date (5). The catalytic component of the nitrogenase system contains two metalloenzymes, dinitrogenase (MoFe protein, for molybdenum nitrogenase) and dinitrogenase reductase (Fe protein). MoFe protein is an α_2_β_2_-heterotetramer with α and β subunits encoded by *nifD* and *nifK* genes, respectively (6). Each αβ-dimer contains two metal clusters: the P cluster ([Fe_8_S_7_]) located at the α/β subunit interface and the iron-molybdenum cofactor ([MoFe_7_S_9_C-homocitrate]) buried within the α-subunit (7–9). The Fe protein is composed of two identical subunits encoded by the *nifH* gene, which are bridged by an [Fe_4_S_4_] cluster to form a holoprotein with two MgATP-binding sites (10). Fe protein is a multifunctional component of the nitrogenase system. It is involved in the maturation of FeMoco and P clusters in the MoFe protein (11). Additionally, Fe protein is an obligate electron donor for MoFe protein under *in vivo* conditions. However, *in vivo* electron donors for Fe proteins are diverse and depend on the lifestyle and metabolism of the host diazotroph (12).

Although only *nifH*, *nifD*, and *nifK* are required to encode the catalytic subunits of nitrogenase, genes encoding components for metal cofactor biosynthesis and accessory proteins for maturation and stabilization of the core enzyme are also necessary for the full activation of nitrogenase (13). Among these, *nifWZ* gene products are involved in stabilizing the apo-MoFe protein and maturation of the P-cluster (14, 15), and *nifUSVENXBQY* gene products are crucial for the biosynthesis of FeMoco for MoFe protein *in vivo* (13, 16). In addition to the [FeS] cluster provided by NifUS, maturation of the Fe protein also depends on the gene product of *nifM* in the model diazotrophic bacteria *Klebsiella oxytoca* and *Azotobacter vinelandii* (17, 18). However, *nifM-like* genes are predominantly found in alpha-, beta-, and gamma-proteobacteria, as well as in a limited number of thermodesulfobacteriota, but are absent in the remaining diazotrophic organisms (19–21). The C-terminal region of NifM features a conserved ppiC-type peptidyl-prolyl isomerase (PPIase) (22). In contrast, the N-terminal portion is less conserved and lacks homology with any known protein domains. Previous research has suggested that NifM may function as a PPIase that catalyzes the isomerization of proline residues between *cis* and *trans* conformations in NifH, specifically targeting the proline residue at position 258 (Pro^258^) in *A. vinelandii* NifH (22). However, the high conservation of seven prolines, including Pro^258^, among NifH proteins from organisms with or without *nifM* raises questions regarding the proposed catalytic function of NifM. The necessity of isomerase function for the maturation of NifH proteins in organisms lacking *nifM* remains unresolved.

In this study, we performed an extensive analysis of the role of NifM within the reconstructed nitrogenase system from *K. oxytoca* in *E. coli*. Mutagenesis and truncation analyses revealed that the C-terminal PpiC isomerase domain of NifM plays a predominant role in the maturation of NifH. Bacterial two-hybrid assays suggested that the N-terminal domain may facilitate the targeting of NifMc to the substrate NifH. Subsequent proline scanning identified Pro^256^, rather than the previously suggested Pro^258^, as more likely to be involved in NifM-directed maturation. A systematic analysis of NifM dependence was conducted on 15 NifHs, including two VnfHs and two AnfHs, from various bacterial sources, demonstrating that the isomerase domain may not be essential for the maturation of NifH in bacteria lacking *nifM*. Protein shuffling experiments indicated that the NifM dependency of NifH may have arisen from whole-protein coevolution, as simple fragment substitutions did not alter this dependence. This study provides novel insights into the function of NifM and its relationship with NifH while also suggesting new directions for future investigations of NifH maturation processes.

## RESULTS

### Functional analysis of the N- and C-terminal domains of NifM

**Fig 1:**
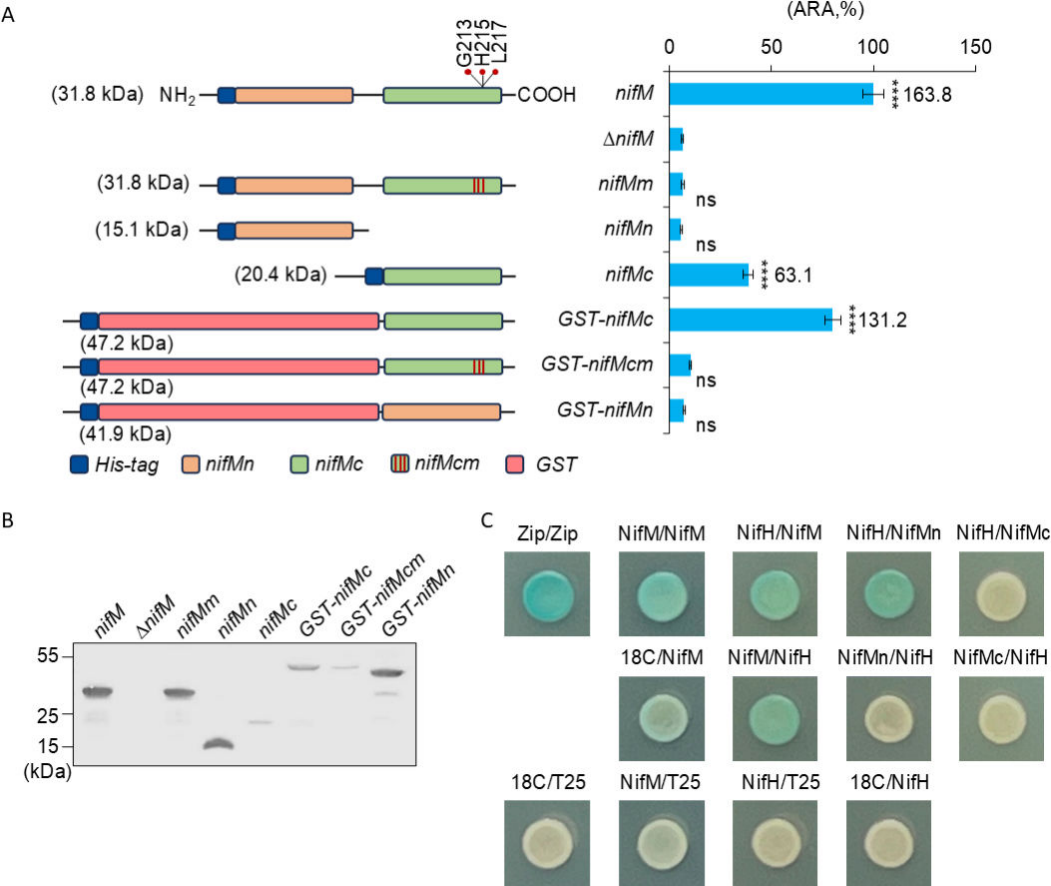
Functional analysis of the N- and C-terminal domains of NifM. (**A**) Mutagenesis analysis to identify functional domains of NifM. The three red lines in the left panel represent the G213A, H215A, and L217A triple mutants within the PpiC domain of NifM. Intact *nifM* or *nifM* mutants were expressed from the *P_tac_* promoter and induced with 400 µM IPTG in *E. coli*. Nitrogenase activity was tested using an acetylene reduction assay, and the absolute values (nM C_2_H_4_/h/OD_600_) for *nifM*, *nifMc,* and *GST-nifMc* are displayed at the top of the bar as the mean of three replicates. The activity observed from intact *nifM* was assigned as 100%, and the error bars represent the mean ± SD of at least three biological replicates. One-way ANOVA was employed to analyze the statistical significance between the *nifM* mutants and the negative control (*∆nifM*); ns, not significant; ****P < 0.0001. (**B**) Western blot assay for the protein levels of NifM variants. Antibodies against His-tags were used. (**C**) BTH analysis of the interaction between NifM and NifH proteins. The negative output for NifMn/NifH may have resulted from an unbalanced protein ratio, as the pUT18C vector harbors a pUC origin with a high copy number, whereas the pKT25 vector has a p15A origin with a medium copy number. Proteins on the left and right sides of the slash represent proteins fused with the 18C fragment or T25 fragment of *Bordetella pertussis* adenylate cyclase, respectively. Zip/Zip: pUT18C-zip/pKT25-zip, assigned as positive control; 18C/T25: pUT18C/pKT25 empty vector, assigned as negative control.

NifM has long been regarded as an isomerase due to the presence of a conserved PpiC-type isomerase domain at its C-terminus. To validate this hypothesis, site-directed mutagenesis was employed to substitute alanine for three highly conserved residues, G213, H215, and L217, which are implicated in the isomerase function of the PpiC-type isomerase, as previously described (23). As anticipated, the mutated NifM failed to restore the acetylene reduction activity (ARA) of the reconstituted nitrogenase system carrying the *nifM* deletion (Fig. 1A). Subsequently, the potential role of the N-terminus of NifM was examined by separately expressing two truncated NifM mutants (NifMn and NifMc) in *E. coli*. The results indicated that NifMn did not restore NifM function (Fig. 1A). In contrast, NifMc, which contains the entire PpiC domain, exhibited only 39% recovery of activity (Fig. 1A). Subsequent western blot analysis revealed that the protein level of NifMc was significantly lower than that of NifM (Fig. 1B). Considering that the observed activity for NifMc might be attributed to reduced protein stability, a GST-NifMc fusion protein was constructed to enhance NifMc stability. The fusion protein appeared to improve protein stability, as evidenced by a substantial increase in GST-nifMc protein levels (Fig. 1B). Additionally, the activity was significantly restored, reaching 80% (Fig. 1A). However, further enhancement of the restored activity was not achieved by increasing the expression of GST-NifMc (Fig. S1), suggesting that NifMn may be essential for full NifM activity. Given that enzymes typically function as polymers, it was hypothesized that NifMn facilitates NifM polymerization. To test this hypothesis, the soluble NifM protein was purified via His-tag affinity purification, and a size exclusion chromatography (SEC) assay was conducted, revealing that the molecular weight corresponding to the peak elution time of NifM was approximately 30 kDa, which closely aligned with the molecular weight of the NifM monomer (Fig. S2). Notably, the possibility of NifMn functioning in polymerization was excluded by the SEC assay. Another possibility is that NifMn is responsible for specifically targeting NifM to NifH, as NifH is a potential substrate of NifM. BTH assay validation supported this hypothesis, demonstrating cross-reactivity between NifM and NifMn with NifH. Conversely, no stable interaction was observed between NifMc and NifH (Fig. 1C).

Through the creation and expression of truncated mutants, along with the execution of protein-protein interaction assays, it was demonstrated that NifMc dominated NifH maturation and that NifMn may facilitate the folding of NifH proteins mediated by NifMc through its binding to NifH.

### Identification of the potential NifM substrate in NifH

**Fig 2:**
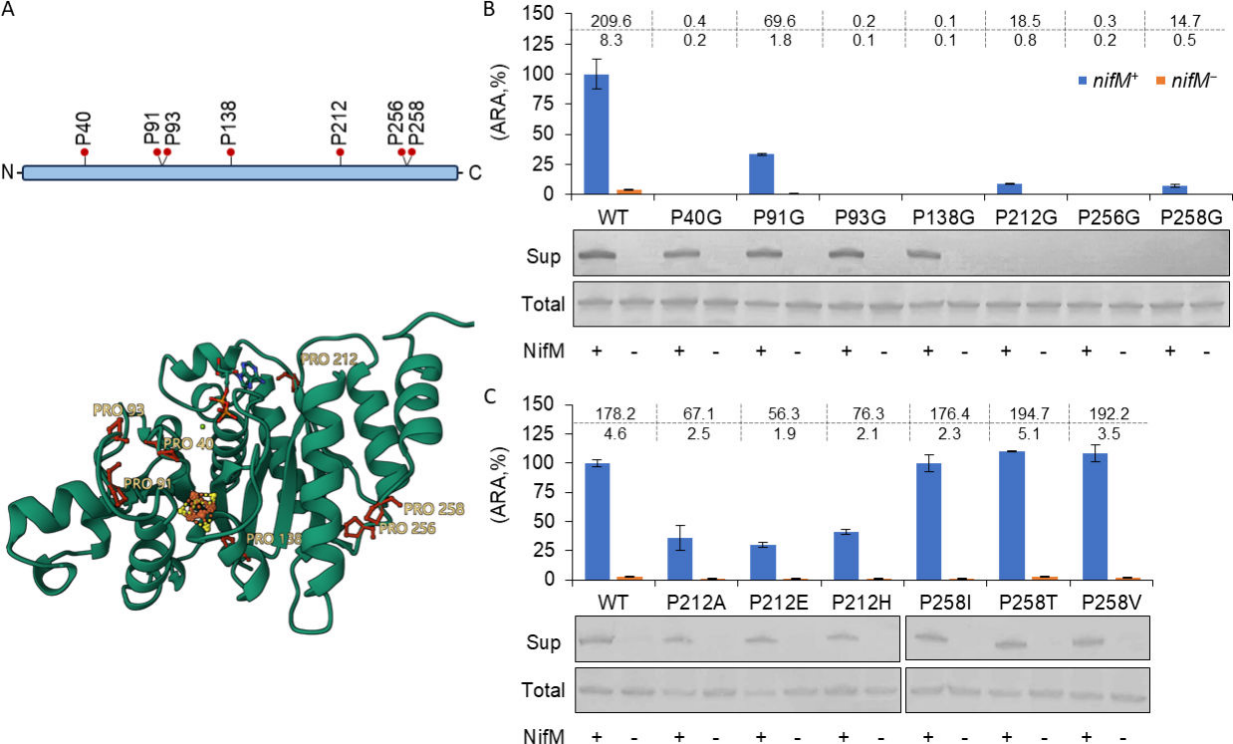
Scanning of the potential site involved in NifM-directed maturation of NifH. (**A**) Distribution of seven conserved proline residues in AvNifH (PDB ID: 1FP6). The side chains of the seven conserved proline residues are displayed as sticks and highlighted in red. (**B** and **C**) Acetylene reduction and protein solubility assays of NifH carrying different mutations. *nifM^+^*/*nifM^−^*, in the presence or absence of *nifM*; WT, wild-type NifH; Sup, soluble proteins from the supernatant fraction; Total, total bacterial cell lysates; +/−, samples prepared from *nifM* plus or minus background, respectively. ARA observed from intact *nifH* under *nifM^+^* conditions was set to 100%, and the absolute values (nM C_2_H_4_/h/OD_600_) are displayed in the table at the top of the bar graph as the mean of three replicates. Error bars represent the mean ± SD of at least three biological replicates.

A previous study posited that Pro^258^ of NifH might act as a substrate for NifM in *A. vinelandii*, with a single mutation replacing the proline residue at position 258 with a serine residue, circumventing the NifM dependence of NifH (22). To examine the broader relevance of this mutation in NifH from other diazotrophs, we incorporated it into *K. oxytoca* NifH (KoNifH). In addition, *A. vinelandii* NifH (AvNifH) and its P258S substitution mutant were cloned and used as a control. Contrary to the previous study, both KoNifH-P258S and AvNifH-P258S mutants showed no detectable activity when *nifM* was absent, as no soluble proteins were detected (Fig. S3A). Meanwhile, only 20%–30% of activities were observed in the presence of *nifM*, due to the decreased soluble fraction compared to their corresponding wild-type NifHs (Fig. S3A). These results indicate that the Pro^258^ site of NifH is unlikely to be involved in NifM-directed maturation. To further explore the potential sites of NifH involved in NifM-directed maturation, we systematically replaced each of the seven conserved proline residues in NifH with glycine (Fig. 2A and B). Four of these mutants, NifH-P40G, -P93G, -P138G, and -P256G, completely lost their ability to restore nitrogenase activity, irrespective of the presence of *nifM* (Fig. 2B). The remaining three mutants, NifH-P91G, -P212G, and -P258G, displayed partial activity but maintained their dependence on NifM (Fig. 2B).

The efficacy of the activity-based system in determining NifM dependence is compromised when mutated NifH loses its capacity to restore nitrogenase activity. In our previous study, we observed that KoNifH solubility in *E. coli* followed a NifM-dependent manner (24). Consequently, solubility could serve as an additional criterion for NifM dependence beyond nitrogenase activity. Given these circumstances, we evaluated the solubility of all seven mutants using antibodies against the His-tag, as all mutants were labeled with a His-tag (MGSSHHHHHHSSG) at the N-terminus. Distinct bands with comparable intensities to those of wild-type NifH were detected for NifH-P40G, -P91G, -P93G, and -P138G in the soluble fraction of the samples when *nifM* was present (Fig. 2B). Although no activity was detected in the NifH-P40G, -P93G, and -P138G mutants (Fig. 2B), we successfully obtained active mutants at these sites that exhibited NifM-dependent activity (Fig. S3C; Table S1). These findings suggest that these sites may contribute to the functionality of NifH in ways that extend beyond the protein folding associated with NifM. Conversely, no detectable amount of soluble protein was observed in the samples prepared from NifH-P212G, -P256G, or -P258G, both in the presence and absence of *nifM*, although approximately the same amount of NifH was observed in total protein samples (Fig. 2B). Saturated libraries were constructed at these three positions to further evaluate NifM dependence. The results showed that no detectable nitrogenase activity was observed when Pro^256^ was replaced with any of the remaining 19 proteinogenic amino acids (Table S2). Subsequent western blot analysis revealed that all NifH-P256 variants were insoluble, regardless of NifM (Fig. S4). In contrast, certain mutants with substitutions at Pro^212^ and Pro^258^ exhibited variable *nifM*-dependent activity. Specifically, NifH-P212A, -P212E, and -P212H recovered over 30% of the wild-type NifH activity, whereas NifH-P258I, -P258T, and -P258V displayed approximately 100% activity (Fig. 2C; Table S2). Moreover, the three most active substitution mutations at both Pro^212^ and Pro^258^ produced detectable amounts of soluble proteins when *nifM* was present (Fig. 2C). Substituting the Pro^258^ residue of AvNifH with a valine residue (AvNifH-P258V) resulted in over 70% activity and a clear band in western blotting for NifH under *nifM^+^* conditions (Fig. S3B). Considering the variations in NifM and the potential coevolutionary relationship between NifM and NifH, it is possible that AvNifM is essential for the optimal functionality of AvNifH mutants. To rule out this possibility, we constructed a plasmid by replacing *KonifM* with *AvnifM*. Subsequently, we evaluated the performance of the AvNifH-P258 mutants in the presence of AvNifM. The activity of AvNifH and its P258 variants exhibited a slight increase; however, there was no significant difference in the ratio between the variants and wild-type NifH under *AvnifM* conditions compared to those under *KonifM* conditions (Fig. S3D).

After thoroughly evaluating the activity and solubility, we hypothesized that the Pro^256^ site of NifH exhibits a higher probability of involvement in NifM-directed maturation than the Pro^258^ site.

### Genetic analysis of the NifM dependence of the Fe protein from diverse origins

**Fig 3:**
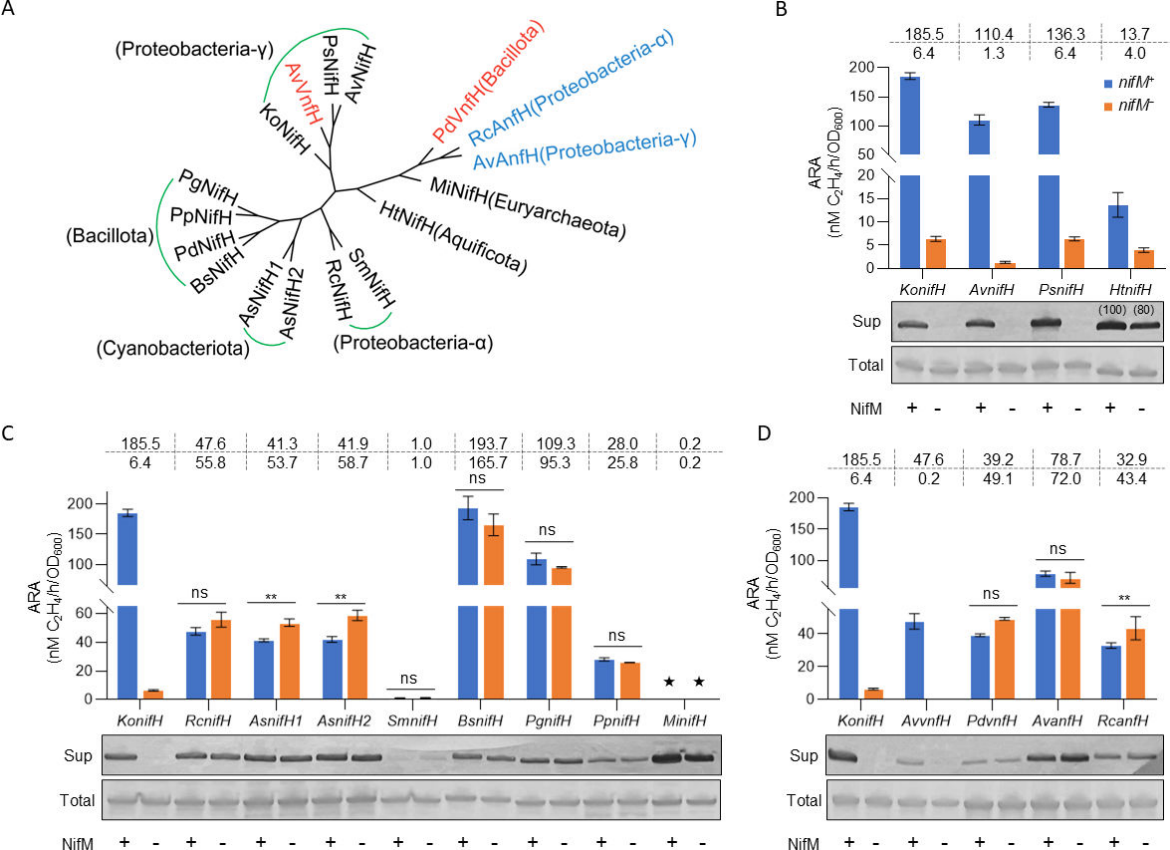
Genetic analysis of iron proteins of diverse origins. (**A**) Phylogenetic associations among selected H proteins, with VnfH and AnfH highlighted in red and blue, respectively. The bacterial phyla corresponding to the origin of the H proteins are noted in parentheses. Acetylene reduction and protein solubility assays of NifM-dependent NifH (**B**), NifM-independent NifH (**C**), VnfH, and AnfH proteins (**D**) from different bacterial origins. The numbers within the brackets in panel **B** above the western bands of MiNifH represent the quantitative data for the western bands, with the values under the *nifM^+^* condition set to 100. ★ in panel **C**, no activity was obtained; Sup, soluble proteins from the supernatant fraction; Total, total bacterial cell lysates; +/−, samples prepared from *nifM* plus or minus background, respectively. In each case, the ARA observed from *nifH/vnfH/anfH* under *nifM^+^* conditions was set to 100%, and the absolute values (nM C_2_H_4_/h/OD_600_) are displayed in the table at the top of the bar graph as the mean of three replicates, and full absolute values are provided in Table S4. Error bars represent the mean ± SD of at least three biological replicates. One-way ANOVA was employed to analyze the statistical significance between the presence or absence of *nifM*; ns, not significant; **P < 0.01.

It is challenging to discern the dependence of NifH on NifM across diverse nitrogen-fixing bacteria that rely solely on primary amino acid sequences. This difficulty arises from the high conservation of seven prolines, including Pro^258^ (the potential NifM target), in nearly all H proteins, including NifH, VnfH, and AnfH from the MoFe, VFe, and FeFe nitrogenase systems (Fig. S5). Furthermore, no additional sequence features of NifH proteins have been identified to elucidate NifM dependence. Our reconstituted *nif* system allowed for easy assessment of NifM dependence on specific bacterial NifH proteins by substituting the *KonifH* gene with the corresponding NifH-coding gene (Fig. S6). Using this approach, we investigated the NifM dependence of NifH from representative nitrogen-fixing bacteria, encompassing 11 NifHs, two VnfHs, and two AnfHs (Fig. 3A; Table S3). Fifteen distinct hybrid nitrogenase systems were engineered by replacing *KonifH* with the coding sequences of NifH, VnfH, and AnfH, all coupled with KoNifDK. The restored nitrogenase activity of each hybrid system was evaluated with and without *nifM*. Consistent with previous findings (25), NifH and VnfH from *A. vinelandii* and NifH from *Pseudomonas stutzeri* exhibited obligate NifM dependence (Fig. 3B and D). Unexpectedly, NifM showed mild inhibitory effects on NifH, VnfH, and AnfH from *Rhodobacter capsulatus, Paenibacillus durus*, and cyanobacteria, as shown by the lower recovered activity in the presence of *nifM* than in its absence (Fig. 3C and D). NifH from *Bacillota* showed limited dependence on NifM, as NifHs from *Bacillus* and *Paenibacillus* exhibited over 85% ARA activity in the absence of *nifM* compared to its presence (Fig. 3C). We found that NifH of *Hydrogenobacter thermophilus* is a nonobligate NifM-dependent type, which can recover approximately 30% ARA activity without *nifM* (Fig. 3B). However, no activity was observed for NifH from *Methanocaldococcus infernus* (Fig. 3C), which may be attributed to the incompatibility of MiNifH with KoNif components, as it has been demonstrated to be incompatible with AvNifDK under *in vitro* conditions (26, 27). We further evaluated the dependence on NifH proteins of diverse origins on NifM by examining their protein solubility (Fig. 3B through D). Western blot analysis revealed that the solubility of most NifH proteins from diverse origins was aligned with their dependence on NifM (Fig. 3B). Notably, NifH from *Rhizobium meliloti*, two VnfHs, and AnfH from *R. capsulatus* exhibited soluble proteins that were not consistent with the total protein levels (Fig. 3C and D), suggesting the involvement of additional proteins necessary for proper folding in the native host. An additional exception is NifH from *H. thermophilus*, which is NifM dependent in terms of activity (Fig. 3B). However, the soluble protein produced only slightly decreased, which was approximately 80% in the absence of *nifM* compared to its presence (Fig. 3B), consistent with the results of a previous study conducted in yeast (21). It is noteworthy that NifHs from thermophiles may exhibit high folding efficiency, as both HtNifH and MiNifH showed high solubility in both *E. coli* and yeast mitochondria (Fig. 3B and C) (21, 26).

### Endogenous PpiC isomerase domain-containing protein is unable to perform like NifM

**Fig 4:**
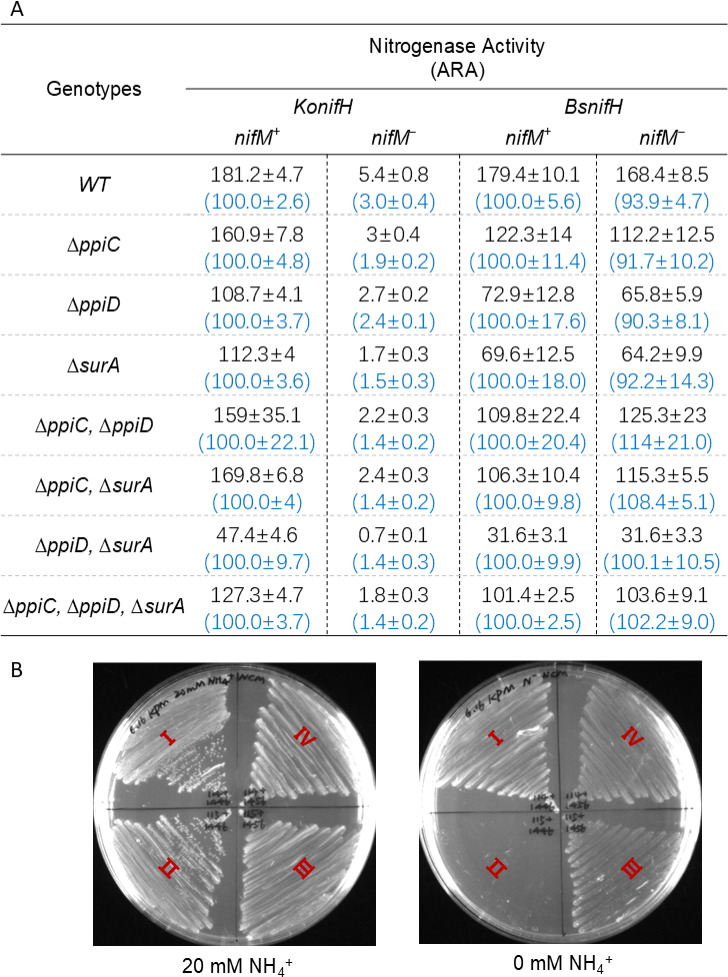
Effect of endogenous PpiC domain-containing proteins on NifM-independent NifH. (**A**) Acetylene reduction assay of the nitrogenase system carrying the *KonifH* and *BsnifH* genes in different bacterial backgrounds. WT, wild-type JM109 strain; *∆ppiC*, *∆ppiD*, and *∆surA*, JM109 strains carrying corresponding gene deletions; *nifM^+^*/*nifM^−^*, in the presence or absence of *nifM*. In each case, the absolute values (nM C_2_H_4_/h/OD_600_) and relative activities of the acetylene reduction assays are displayed, with the absolute values on the top and the relative activities below in brackets. The activities observed under *nifM^+^* conditions were set to 100%, and the error bars represent the mean ± SD of at least three biological replicates. (**B**) Diazotrophic growth assay of the nitrogenase system carrying the *KonifH* and *BsnifH* genes. I, *KonifH*/*nifM^+^*; II, *KonifH*/*nifM^−^*; III, *BsnifH*/*nifM^+^*; IV, *BsnifH*/*nifM^−^*.

Three proteins encoded by *ppiC*, *ppiD*, and *surA* possess a PpiC isomerase domain analogous to that of NifM in *E. coli*. To investigate whether these housekeeping NifM orthologs contribute to the maturation of the NifM-independent NifHs identified in this study, *E. coli* strains deficient in *ppiC*, *ppiD*, and *surA,* both singly and in combination, were generated using lambda-red-directed homologous recombination (28). Subsequently, NifM-independent NifH from *Bacillus* sp. 03113, which can fully substitute for KoNifH in the absence of *nifM*, along with NifM-dependent KoNifH, was selected for further validation. The activities of nitrogenase systems carrying either *KonifH* or *BsnifH* were assessed in single-, double-, and triple-deficient *E. coli* strains. Across all deficient strains, the NifM dependency of KoNifH and BsNifH remained constant, and the nitrogenase activities mediated by KoNifH and BsNifH were reduced to varying degrees (Fig. 4A). This reduction in activity may be attributed to the broad impact of these mutations on cellular physiology, as evidenced by the decreased growth rates in these deficient strains compared to the wild-type strain (Fig. S7). However, in certain instances, the extent of enzyme activity decline did not correspond to the observed decrease in growth, particularly in the *ppiD* and *surA* double-deficient strain. The absolute activity of the nitrogenase systems carrying either KoNifH or BsNifH from the *ppiD* and *surA* double-deficient strain decreased to approximately 20% of that of the wild-type strain (Fig. 4A). However, their maximum growth rate did not significantly fall below that of the wild type, with only a slight delay in the lag phase (Fig. S7). Unlike PpiC, both PpiD and SurA possess an additional extended N-terminal region that functions as a molecular chaperone. SurA is involved in the correct folding and assembly of outer membrane proteins (29). Furthermore, overexpression of PpiD can compensate for the function of SurA, even though PpiD lacks PpiC isomerase function (30, 31). The chaperone functions of PpiD and SurA may affect specific proteins that are closely related to the nitrogen fixation process or directly affect the folding of nitrogenase components. The decrease in nitrogenase activity of BsNifH in the deficient strains was generally more pronounced than that of KoNifH (Fig. 4A). Additionally, a previous study found that the N-terminal region of AvNifM exhibits high structural similarity to a specific region of SurA (20). Therefore, we propose that the N-terminal region of NifM may function as a chaperone that specifically acts on KofNifH, but not on BsNifH, to enhance its ability to support higher activity levels. An unexpected observation was that the absolute nitrogenase activities obtained from the *ppiC*, *ppiD*, and *surA* triple-deficient strain for KoNifH and BsNifH exceeded those observed in the *ppiD* and *surA* double-deficient strain (Fig. 4A). This phenomenon may be attributed to the activation of the latent pathways. Deletion of all genes containing the PpiC isomerase domain results in the activation or upregulation of alternative chaperones, thereby compensating for the functions of these genes.

Acetylene reduction assays are commonly employed to measure nitrogenase activity, owing to their simplicity. However, it is important to note that the ability to reduce acetylene does not necessarily reflect the ability to reduce nitrogen molecules in certain cases. To evaluate whether NifM-independent NifH could support nitrogen assimilation by nitrogenase, diazotrophic growth experiments were carried out using *BsnifH*. In accordance with the results of acetylene reduction assays, BsNifH supported the diazotrophic growth of *E. coli* in the presence and absence of *nifM* (Fig. 4B). In contrast, diazotrophic growth was observed only when *nifM* was present in the case of KoNifH (Fig. 4B).

### Simple fragment substitutions are insufficient to reverse NifM dependency

**Fig 5:**
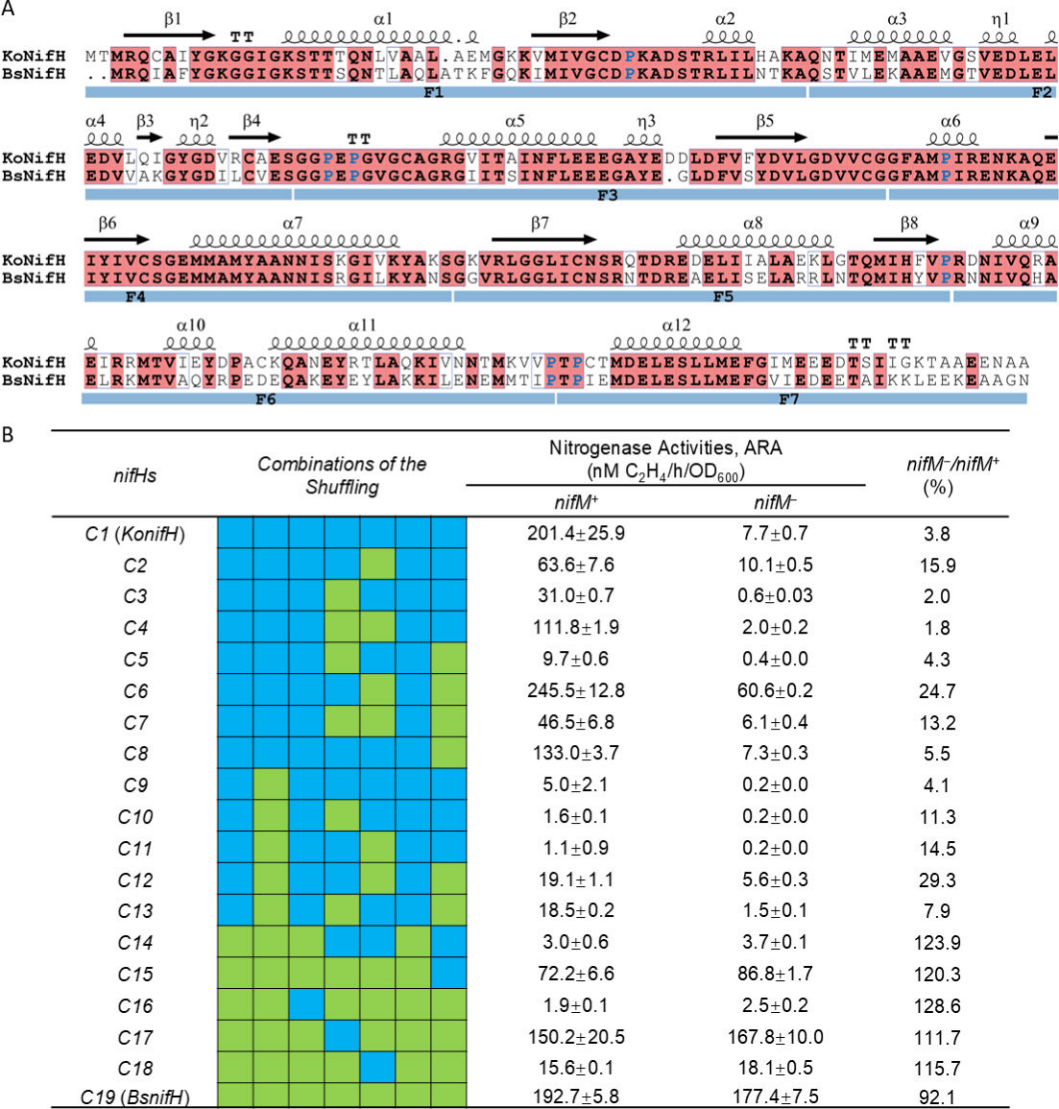
Shuffling analysis of the NifH proteins. (**A**) Schematic showing the division sites of KoNifH and BsNifH. The predicted secondary structure is shown above the sequences, where β represents β-sheet, α represents α-helix, η represents 3_10_-helix, and TT represents β-turn. The seven conserved proline residues are highlighted in blue. The seven divided fragments are marked with blue bars under the sequences labeled F1–F7. (**B**) ARA of the nitrogenase system carrying the chimeric *nifH* genes. Fragments from KoNifH are shown in blue and fragments from BsNifH are shown in green. *nifM^+^*/*nifM^−^*, in the presence or absence of *nifM*. Error bars represent the mean ± SD of at least three biological replicates.

The capacity of BsNifH to substitute for KoNifH in supporting approximately 100% of nitrogenase in a NifM-independent manner suggests a high level of compatibility between BsNifH and KoNif components (Fig. 5B). Therefore, we sought to determine the key features that govern NifM dependence of NifH by constructing chimeric proteins from KoNifH and BsNifH. Both proteins were segmented into seven parts within the conserved coil regions (Fig. 5A) and randomly recombined to generate chimeric NifH. Analysis of the 300 sequenced clones revealed 81 distinct combinations, representing more than 60% of the theoretical possibilities. The ability of these chimeras to restore nitrogenase ARA activity was evaluated under *nifM* plus and minus conditions (Fig. 5B). The *nifM^−^*/*nifM^+^* + was employed as a quantitative measure of NifM dependence, with lower values indicating stronger dependence and higher values suggesting weaker dependence. Notably, 62 of the 81 chimeric NifH proteins completely lost their function (Table S5), whereas 19 chimeras showed detectable activities. Fourteen chimeras had activities higher than that of the wild-type KoNifH (7.7 nM C_2_H_4_/h/OD_600_) in the absence of *nifM*. Among them, several KoNifH-based proteins (containing more KoNifH than BsNifH fragments) exhibited reduced NifM dependence according to the *nifM^−^*/*nifM*^+^ ratio (Fig. 5B). However, it is challenging to determine whether any specific segment significantly influences NifM dependence, as only a limited number of functional chimeras have been identified, and certain fragment substitutions have shown entirely opposite effects in different chimeras (Fig. 5B). Importantly, the C6 chimeric protein exhibited an approximately 20% increase in ARA activity in the presence of NifM (Fig. 5B), suggesting its potential application in the development of highly efficient nitrogenase systems. Our protein shuffling experiments revealed that NifM dependency appears to be a consequence of whole-protein coevolution, as simple fragment substitutions could not fully alter the NifM dependence of NifH. Nevertheless, it remains plausible that the reliance on NifM is affected by a combination of several shorter segments. This hypothesis can be evaluated by further fragmenting NifH for shuffling. However, increasing the number of segments results in a greater number of combinations, thereby complicating the process of obtaining all possible combinations due to the low efficiency of DNA assembly with multiple fragments.

## DISCUSSION

Conservation of *nifM* within the *nifUSV* operon of *K. oxytoca* and *A. vinelandii*, two extensively studied model nitrogen-fixing bacteria, is of particular interest (32, 33). Empirical evidence from diverse genetic and molecular methodologies has corroborated the dependence of NifH on NifM in both the bacterial species (17, 18, 32). This observation led to the hypothesis that NifM is essential for the maturation of all NifH proteins. However, the absence of *nifM* from the *nif* gene cluster or genome of certain diazotrophic bacterial species presents a contradictory scenario (19). Furthermore, the potential proline substrate for NifM is highly conserved in NifH proteins from bacteria that lack *nifM*. These conflicting observations have prompted researchers to reassess the functional roles of NifM. Our investigation demonstrated that the putative isomerase domain of NifM is crucial for NifH maturation (Fig. 1A). Additionally, we elucidated that the N-terminal domain of KoNifM is implicated in targeting the NifM substrate NifH (Fig. 1C). However, it is worth noting that the function identified for the N-terminus of KoNifM may not be conserved in NifM from other nitrogen-fixing bacteria, given the poor conservation of the N-terminal domain (Fig. S8). Concurrently, we cannot exclude other potential functions of the N-terminus, as the PpiC isomerase domain assumes a predominant role in NifH maturation.

Proline, the only proteinogenic amino acid with a constrained phi angle, usually plays a specific role in protein structure (34). Seven prolines were highly conserved in the NifH proteins. Pro^40^, located in the switch I region of NifH, participates in conformational alterations during ATP hydrolysis (35). Pro^91^ and Pro^93^, positioned adjacently, contribute to maintaining the structure necessary for proper [4Fe-4S] cluster binding, with Pro^93^ forming a part of the β-turn. Pro^91^, which lacks association with a specific structure, appears to be less structurally crucial, aligning with the high activity retention observed in its substitution mutant (Fig. 2B). Pro^138^, a component of helix 9, likely plays a vital role in preserving the helix structure and orienting the conserved Arg^140^ for appropriate NifDK interactions (36, 37). Pro^212^ directly engages nucleotides, forming a hydrogen bond at the N6 site of the nitrogenous base (38). This explains why proline substitution with glycine leads to a substantial reduction in activity or functional loss. Intriguingly, Pro^256^ and Pro^258^ lack apparent structural significance or ligand binding involvement from a basic structural standpoint. Initially, we hypothesized that Pro^258^ is the primary target of NifM in NifH. However, the unexpected mutagenic tolerance of this site led us to shift our focus to Pro^256^ (Table S2). Although we were unable to definitively demonstrate the PpiC isomerase activity of NifM, the potential for such activity remains plausible. This inference is supported by the observation that a mutation at the PpiC isomerase activity site resulted in a complete loss of NifM function, which was not attributable to diminished expression of the mutant protein (Fig. 1A and B). Pro^256^ is most likely to be the catalytic site if NifM exhibits PpiC isomerase activity, as all of the remaining six conserved prolines can be replaced with alternative amino acids to achieve NifM-dependent activity (Fig. 2C; Fig. S2). The *trans* form of proline residues exhibits lower “ground state” energy, resulting in approximately 80% *trans*-state proline in unfolded peptides (34), with *cis-trans* interconversion requiring higher ∆G of up to about 80 kJ/mol (39). Notably, all seven proline residues adopted a *trans* form in the final state of both NifM-dependent AvNifH and NifM-independent MiNifH (Fig. S9). Given the substantial energy requirement for *cis-trans* interconversion, we hypothesized that Pro^256^ in NifM-independent NifH would maintain a *trans* state in the unfolded primary state. Conversely, Pro^256^ in NifM-dependent NifH likely assumes a *cis* state when unfolded, which is potentially crucial for NifH folding at a specific transition state. The primary state of Pro^256^ may not be dictated by certain single amino acids or peptides, as substitutions failed to reverse the NifM dependence of NifH (Table S2). However, the possibility remains that Pro^256^ in NifM-dependent NifH initially adopts a *trans* state, undergoing dual NifM-catalyzed conversions: *trans* to *cis* during the transition state, and then reverting to *trans* in the final state. In summary, the precise mechanisms governing the catalytic role of NifM in NifH and the maturation of NifM-independent NifH remain elusive. Elucidating these mechanisms potentially necessitates the observation of the transient phase of NifH protein folding; however, this task is challenging because of the rapid nature of the protein folding process.

The conservation of proline residues extends beyond the NifH proteins to encompass the VnfH and AnfH proteins (Fig. S5). Our preliminary investigations revealed inconsistent NifM dependence for NifH, VnfH, and AnfH within the same bacterial species (Fig. 3), which may offer valuable insights into the evolutionary trajectory of nitrogenases. The restricted distribution of NifM, primarily confined to a subset of Proteobacteria (20), has prompted inquiries regarding its evolutionary origin. One hypothesis posits that NifM co-evolved with NifH; however, as NifH developed NifM independence, the latter was eliminated in the majority of bacterial species. This scenario parallels the evolutionary replacement of plant-type GS by bacterial-type GS, with the former persisting only in a limited number of bacteria closely related to plants (40). An alternative proposition suggests that NifM arises independently and is retained in specific groups of nitrogen-fixing bacteria. This raises questions regarding the potential advantages conferred by NifM-dependent NifH in nitrogenase systems. Beyond its potential role in accelerating proline-limited protein folding, PPI-catalyzed Pro cis-trans isomerization has emerged as a crucial regulatory mechanism in cell growth and signaling (41), influencing enzyme activity, protein stability, and protein-protein interactions. In light of these considerations, NifM-mediated protein folding may facilitate NifH interactions with diverse proteins, given its involvement in multiple aspects of nitrogenase cofactor synthesis and electron transport. Moreover, NifM-mediated allosterism could potentially enhance the efficiency of NifH binding and dissociation with various protein partners.

The nitrogenase system is highly complex and involves a large number of genes. Simplification of this system could enhance the feasibility of constructing functional nitrogenase systems in heterologous hosts. Although previous studies have employed a polyprotein strategy to streamline the nitrogenase system (42, 43), the exploration of more simplified alternatives, such as FeFe nitrogenase systems or the recently discovered NifEN-independent MoFe nitrogenase systems (24, 44), remains a promising avenue. Additionally, from a biodiversity perspective, identifying nitrogenase systems with simplified accessory components will facilitate the heterologous transfer of the nitrogenase system.

## MATERIALS AND METHODS

### Bacterial strains and media

*E. coli* strain JM109 was used for the routine cloning and acetylene reduction assays. JM109-*∆ppiC*, JM109*-∆ppiD*, and JM109*-∆surA* bacterial strains were constructed in a previous study using P1 bacteriophage-directed transduction (24), and double- or triple-deficient strains were constructed using lambda-red-directed homologous recombination based on the single gene deleted strains (28). *E. coli* strain BTH101 was employed for the bacterial two-hybrid assay. *E. coli* strain NCM3722 was used for diazotrophic growth experiments. The KPM medium for the nitrogenase activity assay comprised (per liter) the following: 10.4 g Na_2_HPO_4_, 3.4 g KH_2_PO_4_, 26 mg CaCl_2_·2H_2_O, 30 mg MgSO_4_, 0.3 mg MnSO_4_, 36 mg ferric citrate, 7.6 mg Na_2_MoO_4_·2H_2_O, 10 mg para-aminobenzoic acid, 5 mg biotin, 1 mg vitamin B1, 0.1% casamino acids, and 0.8% (wt/vol) glucose, supplemented with 5 mM ammonium sulfate (KPM-HN) or 0.1% glutamate (KPM-LN). Antibiotics were added to 25 µg/mL chloramphenicol (Cm), 100 µg/mL carbenicillin (Carb), and 25 µg/mL kanamycin (Kan).

### Plasmid construction

Plasmid pKU7017 is a pACYC184 derivative containing all seven σ^54^-dependent *nif* operons from *K. oxytoca* constructed with the BioBrick interface (45). Excision of the *nifHDKTY* operon by *Sna*BI resulted in the pBD114 plasmid, designated as *nifM*^+^. To generate the *nifM^−^* plasmid, the *nifUSVWZM* operon bearing a frame-shifted *nifM* gene was used to substitute the original *nifUSVWZM* operon in pBD114, yielding the pBD115 plasmid. To examine the NifM dependence of various NifH from diverse origins and their corresponding mutations, the pNG1463 plasmid carrying the intact *nifHDKTY* operon with the *nifH* gene replaced by *lacZα* flanking two *Bsa*I sites was employed as a general vector (Fig. S6). In this instance, different *nifH* genes were integrated into pNG1463 using the Golden Gate Assembly method. The *nifH* genes from *H. thermophilus* and *M. infernus* and the *vnfH* gene from *P. durus* were chemically synthesized by Tsingke Biotechnology Company with codons optimized according to the codon bias of *E. coli* (Table S6). The remaining *nifH* genes were directly amplified from the genomes of the corresponding bacteria (Table S6). The construction of shuffled NifH variants from KoNifH and BsNifH involved segmenting the coding sequences of KoNifH and BsNifH into seven distinct parts. Each segment was flanked by type II BsaI restriction sites in an inverse orientation, consisting of a recognition site (GGTCTCA) and a 4-nucleotide cleavage site. The corresponding segments derived from KoNifH and BsNifH were bordered by identical 4-nucleotide cleavage sites. The specificity of the 4-base sticky ends generated by BsaI restriction ensured the correct sequential assembly of the seven fragments at both termini, resulting in the formation of a complete NifH heterozygous gene.

### Acetylene reduction assay

The modified acetylene reduction method was employed to assess nitrogenase activity. To quantify nitrogenase activity of the recombinant *E. coli* JM109 strains, cells were cultured overnight in KPM-HN medium. Subsequently, 80 µL of the cell culture was directly inoculated into 2 mL of KPM-LN medium in 25 mL sealed tubes. Acetylene (2.5 mL) was immediately injected, and the gas phase was analyzed after incubating at 30°C for 16 h using a SHIMADZU GC-2014 gas chromatograph.

### NifM purification and size-exclusion chromatography assay

The *nifM* gene, with a His-tag (MGSSHHHHHHSSG) coding sequence appended to its 5′ end via PCR, was fused to the T7 promoter on a plasmid derived from pET28a. The plasmid was subsequently transformed into *E. coli* BL21(DE3) strain. A single colony was inoculated into 5 mL of liquid LB medium and incubated overnight at 37°C with shaking at 220 rpm. The overnight culture was then diluted 1:100 in 800 mL of fresh liquid LB medium. IPTG was introduced to a final concentration of 50 µM once the OD_600_ reached approximately 0.6, and the culture was incubated for 24 hours at 25°C with shaking at 220 rpm. Bacterial cells were harvested by centrifugation at 12,000 × *g* for 5 minutes at 4°C. The bacterial pellet was washed once with PBS (per liter composed of 8.0 g NaCl, 0.2 g KCl, 1.44 g Na_2_HPO_4_, and 0.24 g KH_2_PO_4_, pH, 7.4), centrifuged, and resuspended in 40 mL of PBS. The cells were lysed by adding 4 mL of TieChui Lysis Buffer (BR0005-02, ACE Biotechnology) for 10 minutes. The lysates were centrifuged at 12,000 g for 1 hour at 4°C, and the supernatant was collected and filtered through a 0.22 µM membrane. The supernatant was then loaded onto a 1 mL Ni-NTA prepacked gravity column (C600791, Sangon Biotech), which had been equilibrated with 10 mL of PBS and washed with 10 mL of PBS plus 20 mM imidazole to remove unbound and non-specifically bound proteins. Subsequently, the NifM proteins were eluted three times using 1 mL of PBS plus 200 mM imidazole. The eluted proteins were concentrated to 0.5 mL and further purified by SEC using a Superdex 200 column (17517501, Cytiva). Samples from different elution peaks were separately collected for further analysis. The molecular weights of proteins from various elution peaks were ascertained by comparing the respective elution volumes of these peaks with those of a standard marker protein provided by the Superdex 200 column. The molecular markers employed included thyroglobulin (669.0 kDa), ferritin (440.0 kDa), aldolase (158.0 kDa), conalbumin (75.0 kDa), ovalbumin (44.0 kDa), carbonic anhydrase (29.0 kDa), and ribonuclease A (13.7 kDa). These markers corresponded to elution volumes of 8.92, 10.13, 12.28, 13.73, 14.68, 16.06, and 17.44 mL, respectively, as calculated from the manual of the Superdex 200 column (Fig. S2).

### Bacteria two-hybrid assay

Each protein-coding gene was initially cloned into pUT18C and pKT25 vectors using *Xba*I and *Eco*RI double restriction to form an in-frame fusion to the T18 and T25 fragments of *Bordetella pertussis* adenylate cyclase (46). Plasmids were subsequently transformed into the BTH101 strain, and individual colonies were selected and precultured in LB medium with 200 µM IPTG for 48 h. Next, 2 µL of the bacterial culture was inoculated onto solid LB medium containing 50 µg/mL 5-bromo-4-chloro-3-indolyl β-D-galactopyranoside (X-gal). After incubation at 30°C for 12 h, the interaction is indicated by the blue color produced by LacZ.

### Diazotrophic growth assay

Diazotrophic growth experiments were performed as described previously (43). Briefly, individual colonies of *E. coli* NCM3722 strains harboring the reconstructed plasmids were isolated and inoculated onto KPM-NN plates (without the addition of an exogenous nitrogen source). Subsequently, the plates were transferred to a 2.5 L anaerobic jar equipped with anaerobic gas-generating sachets and an oxygen indicator. The anaerobic jars were promptly sealed and incubated at 30°C for a duration of 3–4 days.

### Western blot

For western blot analysis, samples were collected immediately after nitrogenase activity testing. To prepare the soluble fraction of the protein samples, bacterial cells were resuspended in PBS and lysed using an ultrasonic crusher. The resulting cell lysates were centrifuged at 20,000 × *g* for 10 min at 4, and the supernatant was collected as soluble protein and supplied with an appropriate amount of 5× SDS buffer. To prepare total protein, the bacterial cells were resuspended in PBS and lysed directly with 5× SDS buffer. Approximately 30–40 μg of protein was loaded on 10% SDS-polyacrylamide gels with 6 µL of PageRuler Prestained Protein Ladder as a marker. Proteins on the gels were subsequently transferred to polyvinylidene fluoride (PVDF) membranes using iBolt 2. The membranes were blocked with 5% skim milk in PBS. Antibodies against His-tag were used at a dilution of 1:1,000. The secondary antibody, goat anti-mouse IgG-HRP, was used at a 1:2,000 dilution. Development was performed using two different methods: employing an enhanced chemiluminescent substrate for HRP and captured by an automatic chemiluminescence imaging analysis system, or directly developed on PVDF membranes using the DAB Chromogen/HRP Substrate Kit.

## Data Availability

All the data are available in the main text and supplemental material.

## SUPPLEMENTAL MATERIAL

Supplemental material mbio.02642-25-s0001.docx Figures S1-S9 and Tables S1-S6.

ASM does not own the copyrights to Supplemental Material that may be linked to, or accessed through, an article. The authors have granted ASM a non-exclusive, world-wide license to publish the Supplemental Material files. Please contact the corresponding author directly for reuse.
