# Supplementary material for: Genetic analysis of the NifM dependence of the nitrogenase iron proteins
Source: mBio. 2025 Oct 31;16(12):e02642-25. doi: 10.1128/mbio.02642-25 (PMC12691677; doi:10.1128/mbio.02642-25)
Supplement: Supplemental material — Figures S1-S9 and Tables S1-S6. [file mbio.02642-25-s0001.docx]

**Supplementary information for**

**Genetic analysis of the NifM dependence of the nitrogenase iron proteins**

Zhuoting Xie^1*^, Shuyi Cai^1*^, Haoyang Chen^1*^, Shuyuan Kong^2^, Letian Tang^3^, Yi-Ping Wang^4^, Jianguo Yang^1,4#^

^1^ State Key Laboratory of Gene Function and Modulation Research, School of Advanced Agricultural Sciences, Peking University, Beijing 100871, China;

^2^ College of Plant Protection, China Agricultural University, Beijing 100871, China;

^3^ College of Biological Sciences and Technology, Beijing Forestry University, Beijing 100871, China;

^4^ Yazhouwan National Laboratory, Sanya 572025, Hainan, China

^#^To whom correspondence should be addressed. Email: [yangjg@pku.edu.cn](mailto:yangjg@pku.edu.cn)

^*^ These authors contributed equally to this work


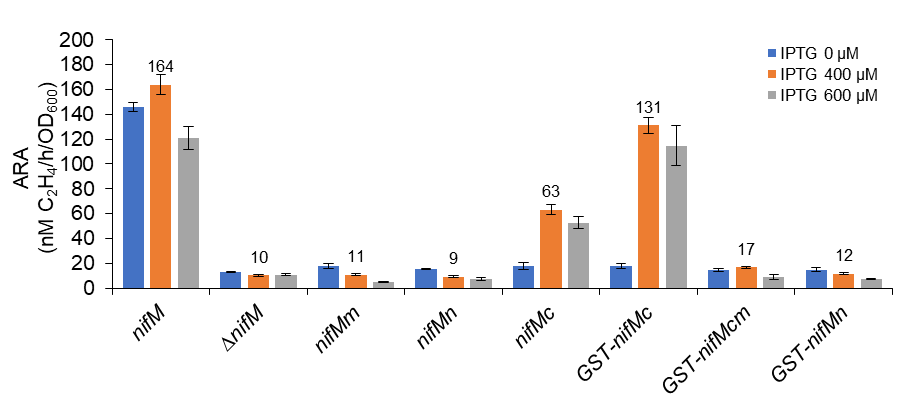


**Figure S1. Functional assay of NifM variants.** Intact *nifM* or *nifM* mutants were expressed from the *P_tac_* promoter and induced with 0, 400, or 600 μM IPTG in *E. coli*. Nitrogenase activity was tested using an acetylene reduction assay, and the absolute values (nM C_2_H_4_/h/OD_600_) obtained from 400 μM IPTG induction are displayed at the top of the bar as the mean of three replicates. Error bars represent the mean ± SD of at least three biological replicates.


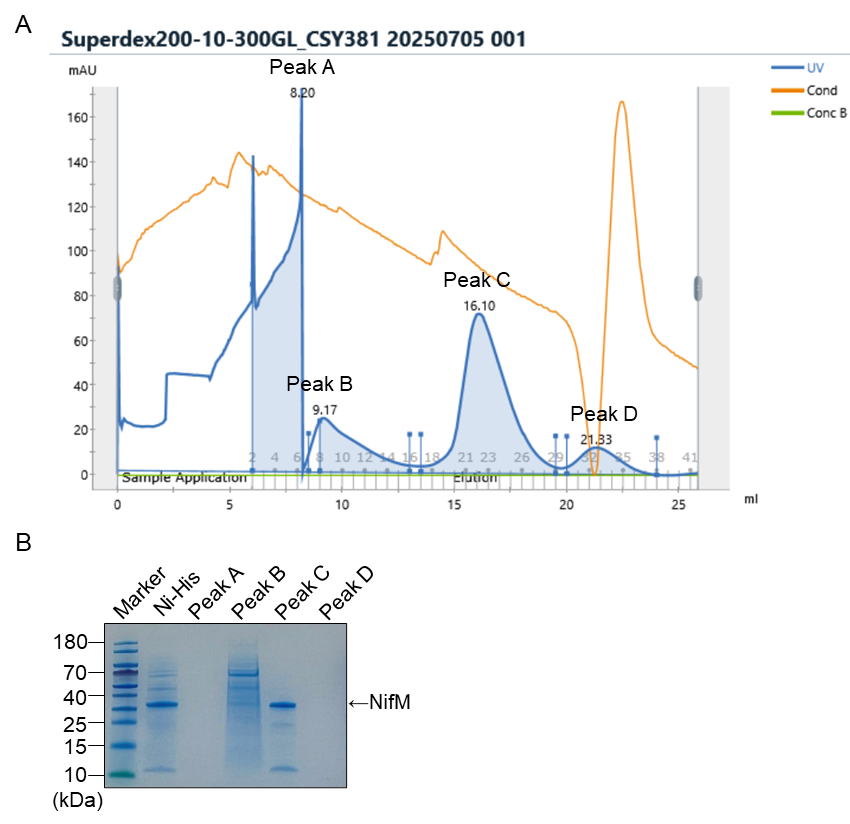


**Figure S2. Size-exclusion chromatography assay of the NifM. (A)** Elution curve for the size-exclusion chromatography. Four elution peaks were labeled as peak A to D respectively. **(B)** SDS-PAGE analysis of the purified NifM protein. Ni-His, original NifM proteins purified from Ni affinity purification; Peak A to D, samples collected from corresponding elution peaks in (A). The elution peak of NifM was observed at 16.10 mL, closely aligning with the elution volume of the standard protein carbonic anhydrase (29.0 kD, 16.06 mL) and situated between those of ovalbumin (44.0 kD, 14.68 mL) and ribonuclease A (13.7 kD, 17.44 mL).


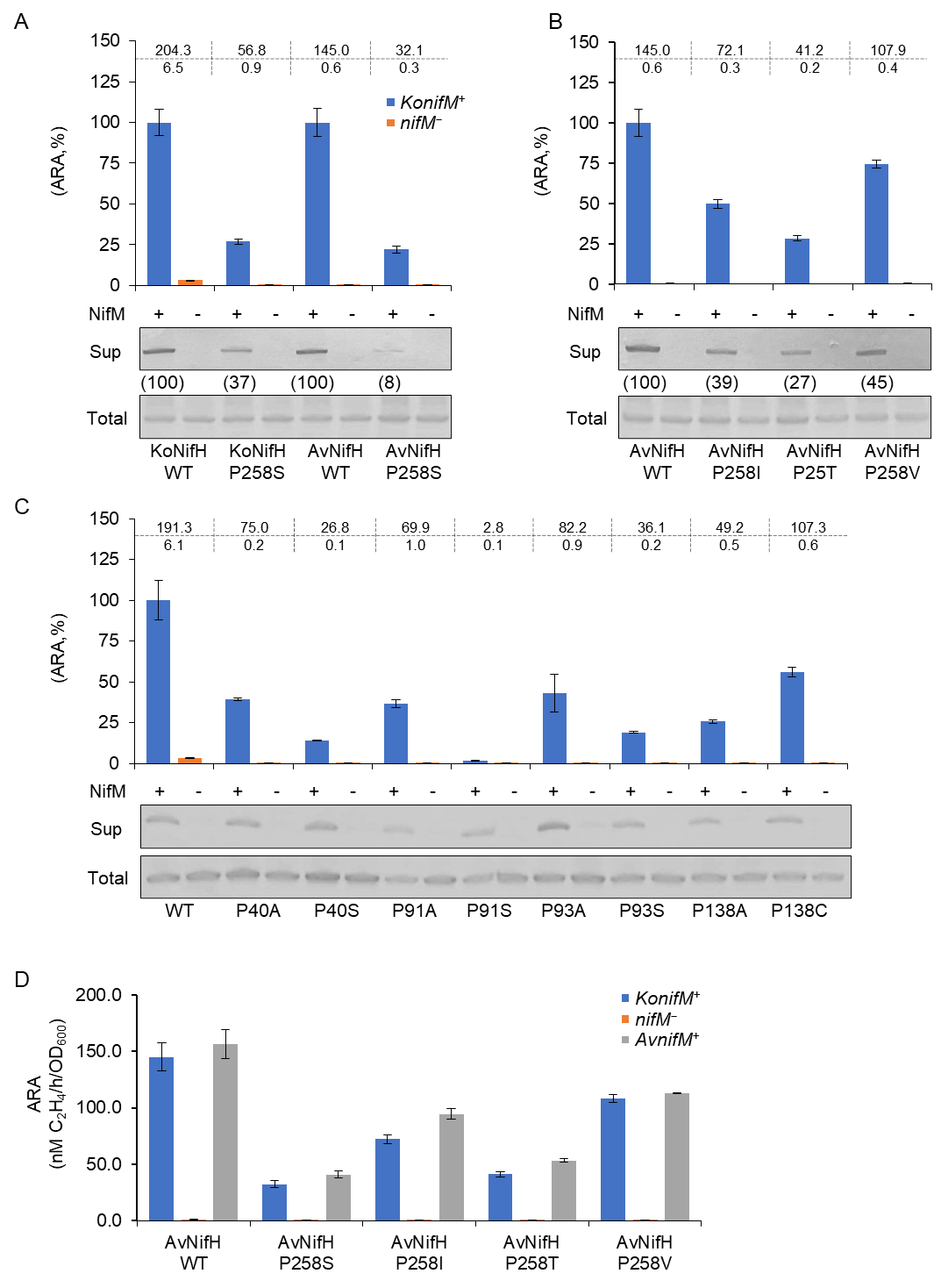


**Figure S3. Functional analysis of the KoNifH and AvNifH mutants.** Acetylene reduction and protein solubility assays for KoNifH P258 variants **(A)**, AvNifH P258 variants **(B)**, KoNifH P40, P91, P93, and P138 variants **(C)**. Acetylene reduction assays for AvNifH P258 variants in the presence of *AvnifM* **(D)**. The acetylene reduction activity (ARA) observed for each intact *nifH* under the *KonifM^+^* conditions was set to 100%, and the absolute values (nM C_2_H_4_/h/OD_600_) are displayed in the table at the top of the bar graph as the mean of three replicates in (A), (B), and (C). *KonifM^+^/nifM^−^*, activity obtained in the presence or absence of *KonifM*; *AvnifM^+^* activity obtained in the presence of *AvnifM* and error bars represent the mean ± SD of at least three biological replicates. Anti-His tag antibody was used to detect the protein levels of NifHs and their variants. Ko, *K. oxytoca*; Av, *A. vinelandii*; WT, wild-type NifH; Sup, soluble proteins from the supernatant fraction; Total, total bacterial cell lysates; +/−, samples prepared from *nifM* plus or minus background, respectively.


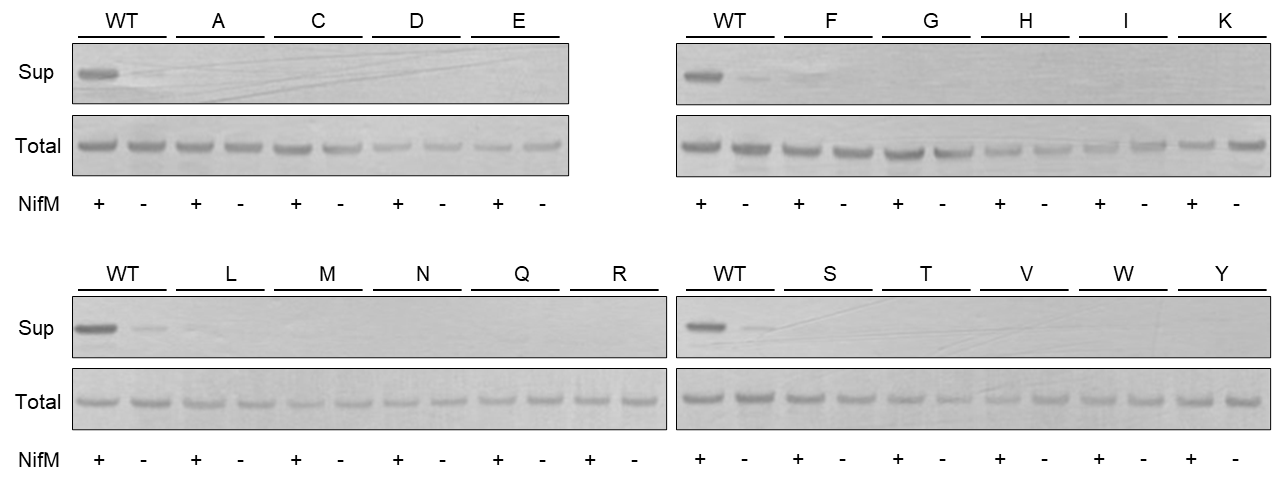


**Figure S4. Solubility assay of KoNifH P256 variants.** Western blot analysis of the protein levels of KoNifH P256 variants with antibodies against the His tag. WT, wild-type NifH; Letters on the top of the western bands represent one-letter abbreviations of the amino acid used for substitute the original proline; Sup, soluble proteins from the supernatant fraction; Total, total bacterial cell lysates; +/−, samples prepared from *nifM* plus or minus background, respectively.


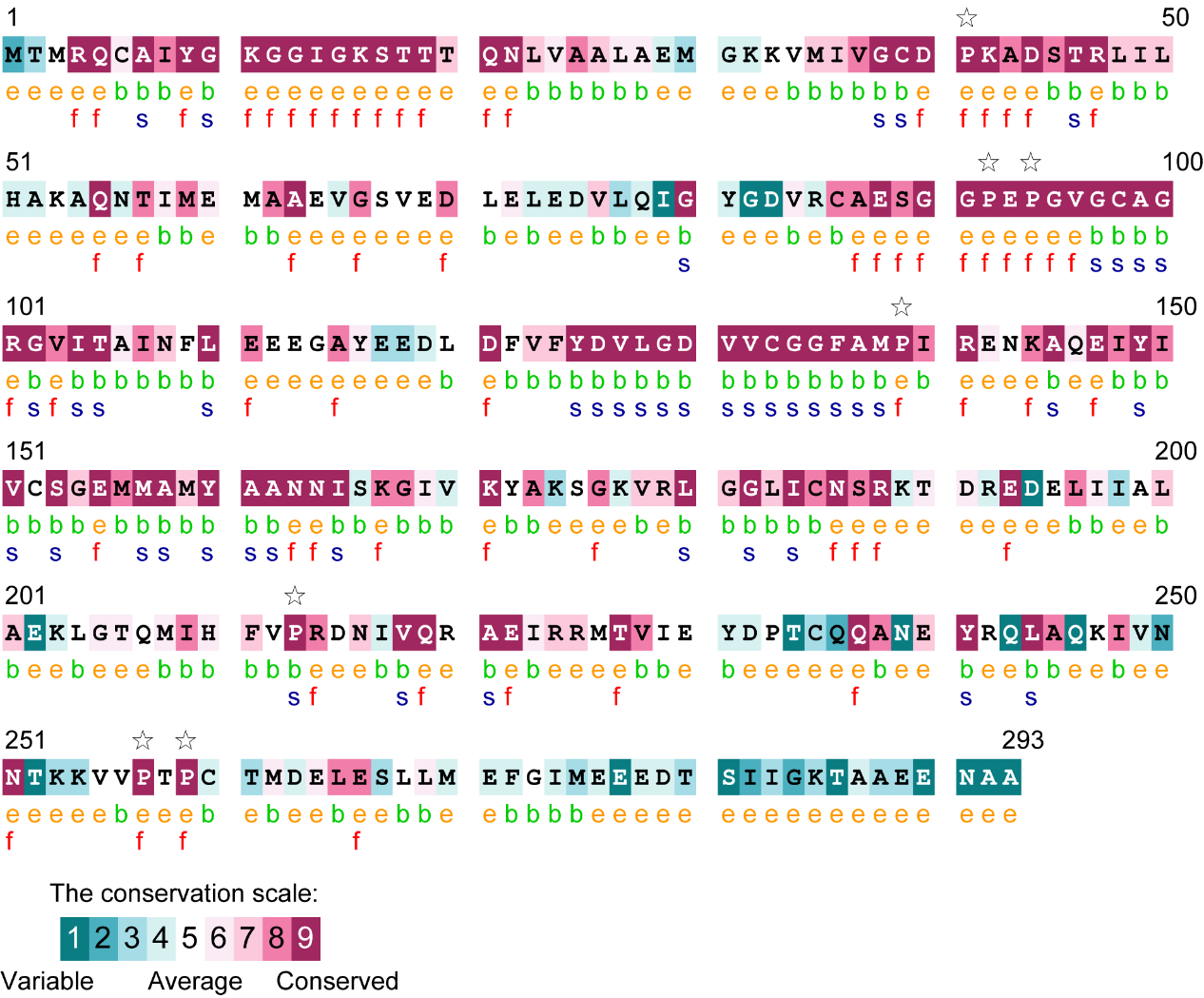


**Figure S5. Sequence conservation analysis of NifH/VnfH/AnfH proteins.** A total of 90 H protein sequences, including 76 NifH proteins, four VnfH proteins, and 10 AnfH proteins, were used to analyze the conservation of each amino acid site using the ConSurf web server (3). The conservation scale of amino acids was classified into nine levels, numbered 1-9. High conservation relative to high values. e, exposed residues; b, buried residues; f, highly conserved and exposed residues; s, highly conserved and buried residues. Seven conserved proline residues are highlighted with star marks.


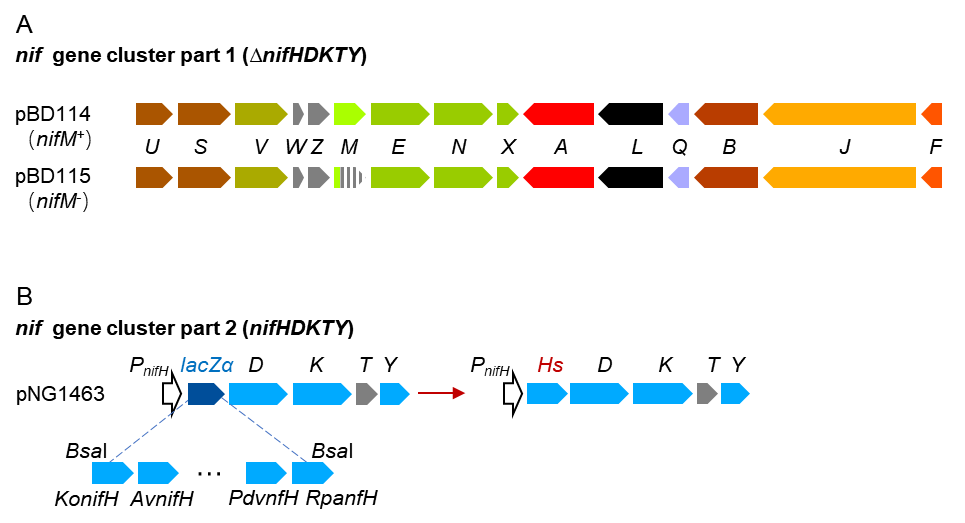


**Figure S6. Schematic** **representation of the plasmids.** **(A)** Schematic showing the gene arrangement of *K.* *oxytoca nif* gene cluster part 1. The pBD114 plasmid carrying the intact *nifM* gene was assigned as *nifM^+^*, and the pBD115 plasmid carrying the frame-shifted *nifM* gene was assigned as *nifM^−^* when co-transformed with *nif* gene cluster part 2 to form a complete nitrogen fixation system. **(B)** Schematic showing the general vector pNG1463 used to host different *nifH* mutants or *nifH* genes from different origins to generate different *nif* gene cluster part2. The *lacZα* was flanked with two *Bsa*I restriction sites and replaced by *nifH* genes with the Golden Gate assembly.


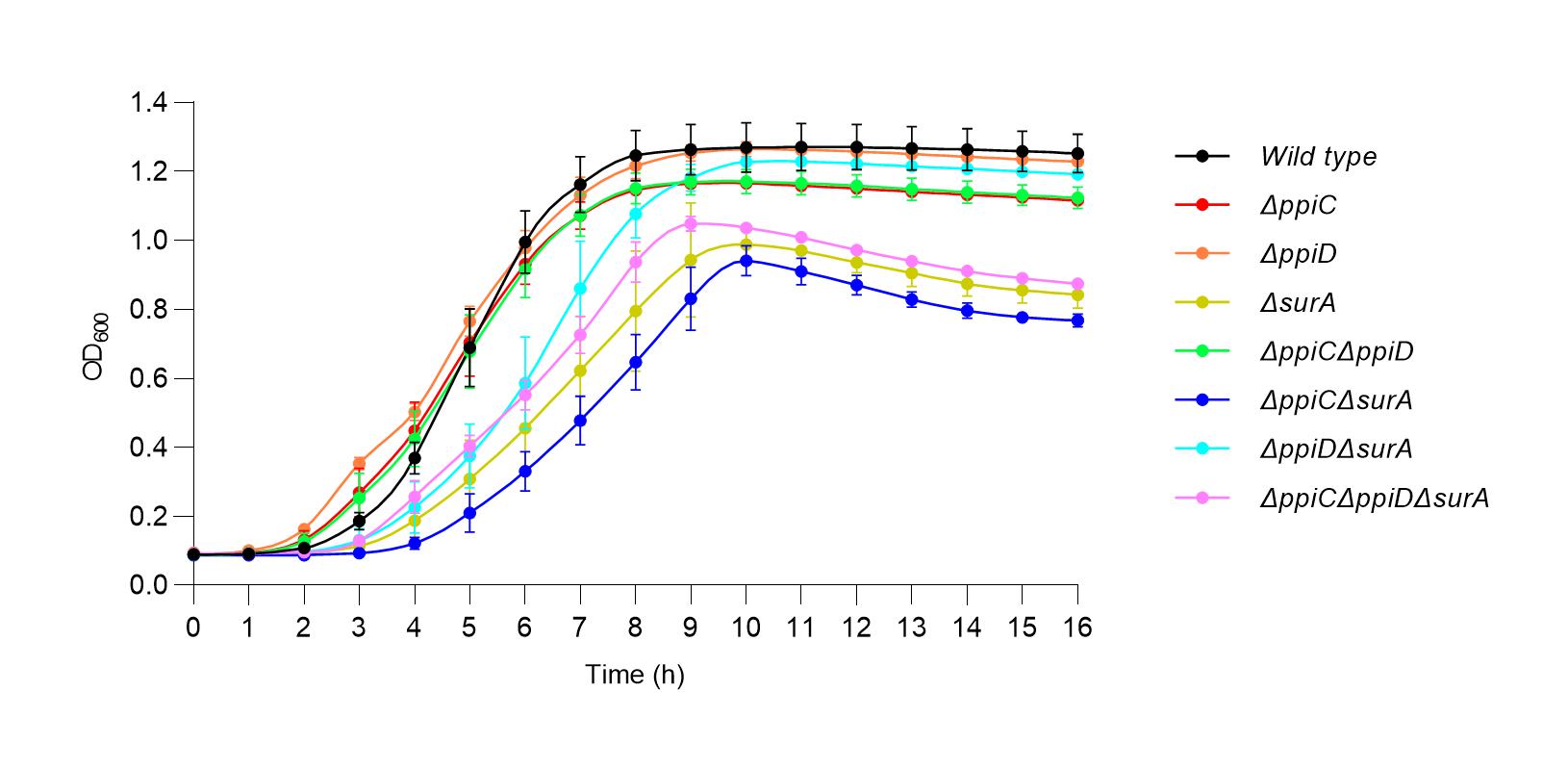


**Figure S7. Growth curves of *E. coli* JM109 gene-deletion mutants.** Absorbance values at 600 nm were measured every hour (h) to monitor real-time biomass accumulation. WT, wild-type JM109 strain; *∆ppiC*, *∆ppiD*, and *∆surA*, JM109 strains carrying corresponding gene deletions. Error bars represent the mean ± SD of three biological replicates.


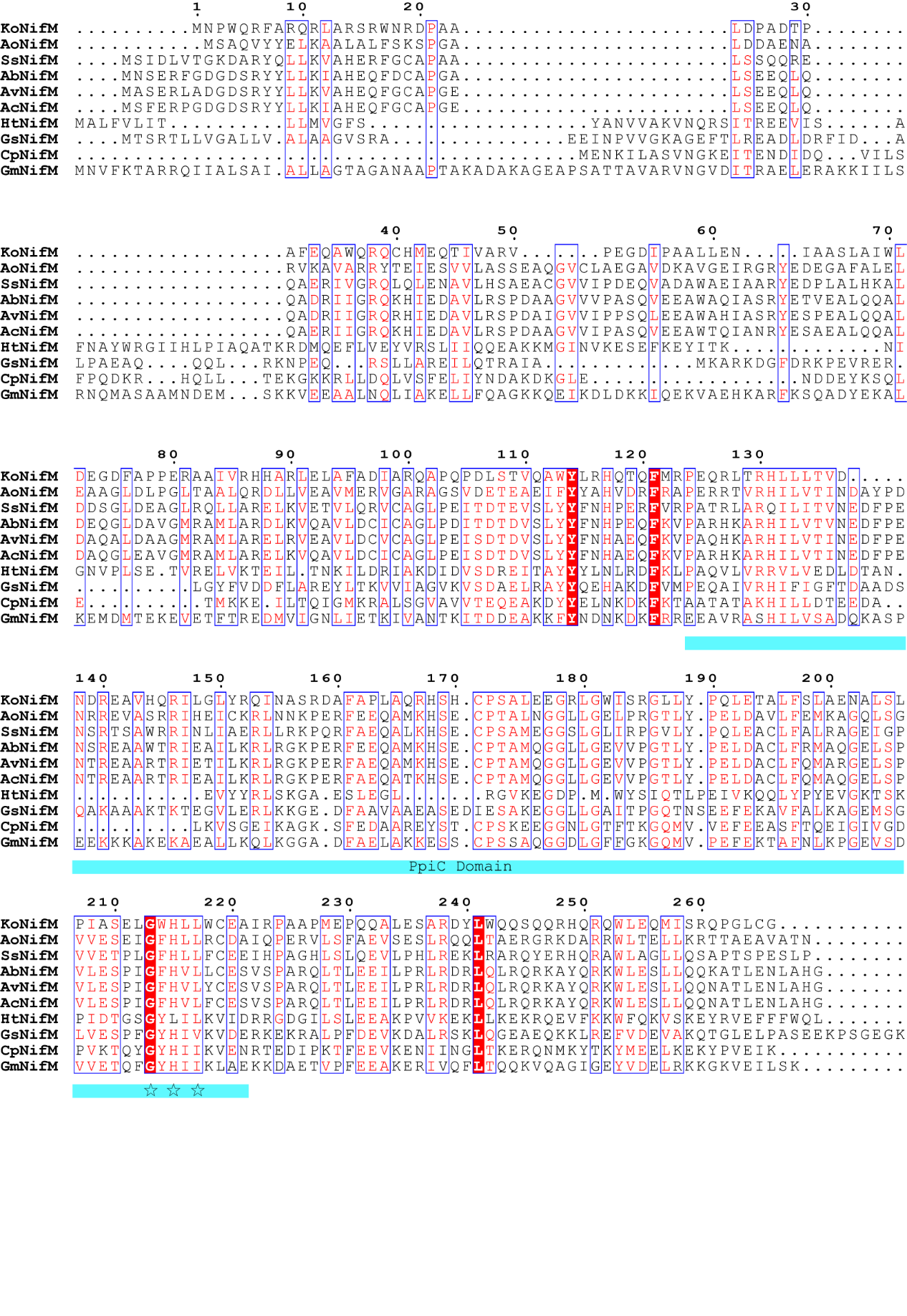


**Figure S8. Sequence alignment of NifM proteins isolated from representative bacteria.** A total of 10 NifM protein sequences were aligned using the online too on Uniprot web server (https://www.uniprot.org/align), and aligned results were subsequently displayed with the ESPript 3.0 (4). The PpiC domain is marked with a cyan bar under the sequence. Three key residues, G213, H215, and L217, associated with the isomerase function of NifM, are highlighted with stars. Ko, *K. oxytoca*; Ao, *Azoarcus olearius*; Ss, *Stutzerimonas stutzeri*; Ab, *Azotobacter beijerinckii*; Av, *A. vinelandii*; Ac, *Azotobacter chroococcum*; Ht, *H. thermophilus*; Gs, *Geobacter sulfurreducens*; Cp, *Clostridium pasteurianum*; Gm, *Geobacter metallireducens*.

**
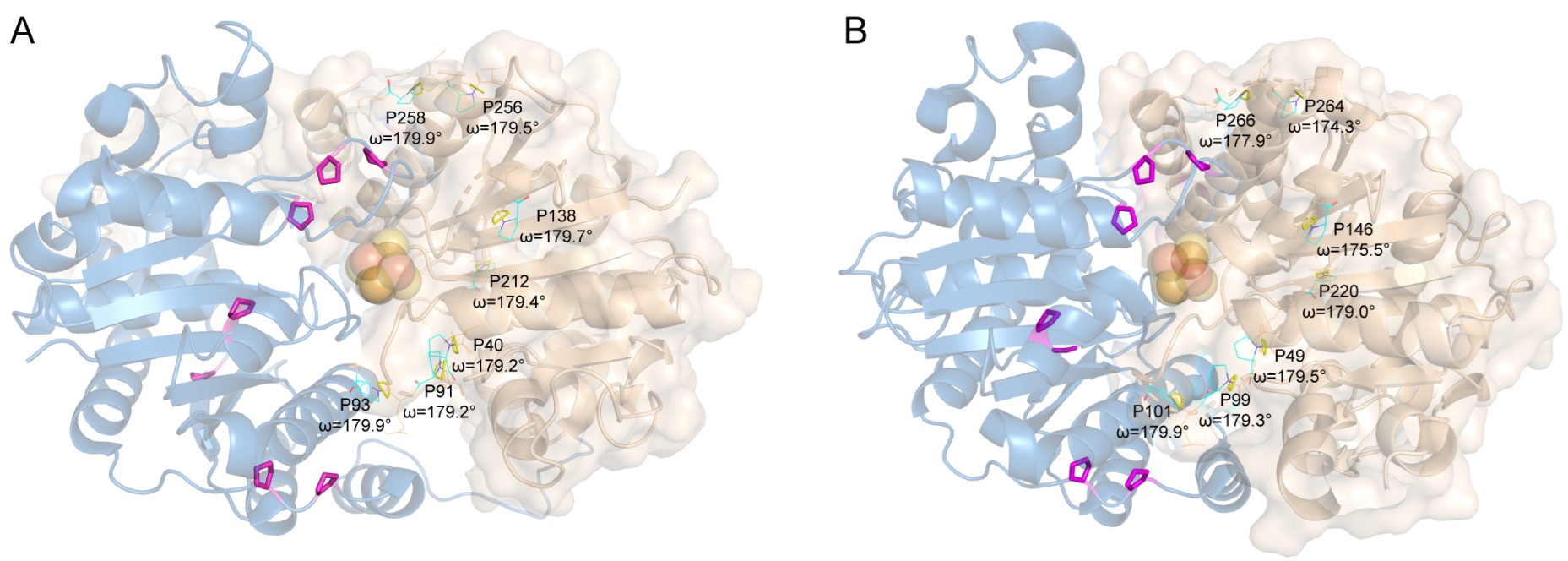
**

**Figure S9. Schematic** **representation of AvNifH and MiNifH structures. (A)** Structure of *A. vinelandii* NifH (PDB ID:1G5P)(5). **(B)** Structure of *M. infernus* NifH (PDB ID:8Q5W)(6). The prolines are labeled in purple with side chain displays in sticks, and the dihedral angles (ω) of the seven conserved prolines are shown.

**Table S1. Relative acetylene reduction activity of saturated mutants at positions P40, P91, P93, and P138.** Letters in the leftmost column represent one-letter abbreviations for the 20 amino acids. The activity observed for wild-type *nifH* under *nifM^+^* conditions was set to 100% (the absolute value is 191.3±22.7 nM C_2_H_2_/h/OD_600_). *nifM^+^* and *nifM^−^*, activity obtained in the presence or absence of *nifM*, and error bars represent the mean ± SD of at least three biological replicates.

| Amino acid  Substituted | P40 | | P91 | | P93 | | P138 | |
| --- | --- | --- | --- | --- | --- | --- | --- | --- |
|  | *nifM^+^* | *nifM^−^* | *nifM^+^* | *nifM^−^* | *nifM^+^* | *nifM^−^* | *nifM^+^* | *nifM^−^* |
| A | 39.2±0.8 | 0.1±0.0 | 36.6±2.3 | 0.5±0.0 | 43.0±11.4 | 0.5±0.1 | 25.7±1.0 | 0.2±0.1 |
| C | 0.2±0.0 | 0.1±0.0 | 0.7±0.6 | 0.1±0.0 | 1.6±0.0 | 0.1±0.0 | 56.1±3.1 | 0.3±0.2 |
| D | 0.1±0.0 | 0.1±0.0 | 0.1±0.0 | 0.1±0.0 | 0.1±0.0 | 0.1±0.0 | 0.1±0.0 | 0.1±0.0 |
| E | 0.1±0.0 | 0.1±0.0 | 0.1±0.0 | 0.1±0.0 | 0.1±0.0 | 0.1±0.0 | 0.3±0.0 | 0.2±0.0 |
| F | 0.3±0.1 | 0.1±0.0 | 0.1±0.0 | 0.1±0.0 | 0.1±0.0 | 0.1±0.0 | 0.1±0.0 | 0.1±0.0 |
| G | 0.2±0.0 | 0.1±0.0 | 20±1.2 | 0.2±0.0 | 0.1±0.0 | 0.1±0.0 | 0.1±0.0 | 0.1±0.0 |
| H | 0.1±0.0 | 0.1±0.1 | 0.1±0.0 | 0.1±0.0 | 0.1±0.0 | 0.1±0.0 | 0.1±0.0 | 0.1±0.0 |
| I | 0.1±0.0 | 0.1±0.0 | 0.1±0.0 | 0.1±0.0 | 0.1±0.0 | 0.1±0.0 | 0.1±0.0 | 0.1±0.0 |
| K | 0.1±0.0 | 0.1±0.0 | 0.1±0.0 | 0.1±0.0 | 0.1±0.0 | 0.1±0.0 | 0.1±0.0 | 0.1±0.0 |
| L | 0.1±0.0 | 0.1±0.0 | 0.1±0.0 | 0.1±0.0 | 0.1±0.0 | 0.1±0.0 | 0.1±0.0 | 0.1±0.0 |
| M | 0.1±0.0 | 0.1±0.0 | 0.2±0.0 | 0.1±0.0 | 0.1±0.0 | 0.1±0.0 | 1.0±0.0 | 0.1±0.0 |
| N | 0.1±0.0 | 0.1±0.0 | 0.1±0.0 | 0.1±0.0 | 0.1±0.0 | 0.1±0.0 | 0.1±0.0 | 0.1±0.0 |
| **P (WT)** | 100.0±11.9 | 3.2±0.2 | 100.0±11.9 | 3.2±0.2 | 100.0±11.9 | 3.2±0.2 | 100.0±11.9 | 3.2±0.2 |
| Q | 0.1±0.0 | 0.1±0.0 | 0.1±0.0 | 0.1±0.0 | 0.2±0.0 | 0.1±0.0 | 0.1±0.0 | 0.1±0.0 |
| R | 0.1±0.0 | 0.1±0.0 | 0.1±0.0 | 0.1±0.0 | 0.1±0.0 | 0.1±0.0 | 0.1±0.0 | 0.1±0.0 |
| S | 14.0±0.2 | 0.1±0.0 | 1.5±0.2 | 0.1±0.0 | 18.9±0.6 | 0.1±0.0 | 0.1±0.0 | 0.1±0.0 |
| T | 0.1±0.0 | 0.1±0.0 | 0.1±0.0 | 0.1±0.0 | 0.1±0.0 | 0.1±0.0 | 0.2±0.0 | 0.1±0.0 |
| V | 0.1±0.0 | 0.1±0.0 | 0.1±0.0 | 0.1±0.0 | 1.8±0.1 | 0.1±0.0 | 0.4±0.1 | 0.1±0.0 |
| W | 0.1±0.0 | 0.1±0.0 | 0.1±0.0 | 0.1±0.0 | 0.1±0.0 | 0.1±0.0 | 0.1±0.0 | 0.1±0.0 |
| Y | 0.1±0.0 | 0.1±0.0 | 0.1±0.0 | 0.1±0.0 | 0.1±0.0 | 0.1±0.0 | 0.1±0.0 | 0.1±0.0 |

**Table S2.** **Relative acetylene reduction activity of saturated mutants at positions P212, P256, and P258.** Letters in the leftmost column represent one-letter abbreviations for the 20 amino acids. The activity observed for wild-type *nifH* under *nifM^+^* conditions was set to 100% (the absolute value is 178.2±4.6 nM C_2_H_2_/h/OD_600_ for P212 group, 185.2±6.1 nM C_2_H_2_/h/OD_600_ for P256 group, and 176.8±8.8 nM C_2_H_2_/h/OD_600_ for P258 group respectively). *nifM^+^* and *nifM^−^*, activity obtained in the presence or absence of *nifM*, and error bars represent the mean ± SD of at least three biological replicates.

| Amino acid  Substituted | P212 | | P256 | | P258 | |
| --- | --- | --- | --- | --- | --- | --- |
|  | *nifM^+^* | *nifM^−^* | *nifM^+^* | *nifM^−^* | *nifM^+^* | *nifM^−^* |
| A | 36.2±10.8 | 1.4±0.0 | 0.1±0.0 | 0.1±0.0 | 30.2±7.1 | 0.4±0.1 |
| C | 9.9±0.0 | 0.3±0.0 | 0.1±0.0 | 0.1±0.0 | 75.5±4.3 | 1.5±0.0 |
| D | 13.4±0.3 | 1.2±0.1 | 0.1±0.0 | 0.1±0.0 | 16.0±0.6 | 0.3±0.0 |
| E | 30.4±2.0 | 1.0±0.5 | 0.1±0.0 | 0.1±0.0 | 23.1±2.6 | 0.6±0.1 |
| F | 0.3±0.1 | 0.1±0.0 | 0.1±0.0 | 0.1±0.0 | 2.6±0.5 | 0.1±0.0 |
| G | 9.4±0.3 | 0.6±0.1 | 0.4±0.0 | 0.1±0.0 | 6.5±0.3 | 0.2±0.0 |
| H | 41.2±2.2 | 1.1±0.0 | 0.1±0.0 | 0.1±0.0 | 2.2±0.2 | 0.1±0.0 |
| I | 0.1±0.0 | 0.1±0.0 | 0.1±0.0 | 0.1±0.0 | 99.8±7.3 | 1.3±0.0 |
| K | 26.4±0.0 | 0.4±0.1 | 0.1±0.0 | 0.1±0.0 | 1.1±0.0 | 0.1±0.0 |
| L | 0.5±0.0 | 0.1±0.0 | 0.1±0.0 | 0.1±0.0 | 27.9±0.3 | 0.5±0.0 |
| M | 2.5±0.2 | 0.1±0.0 | 0.1±0.0 | 0.1±0.0 | 9.9±0.8 | 0.3±0.0 |
| N | 24.8±0 | 0.9±0.1 | 0.1±0.0 | 0.1±0.0 | 12.3±0.1 | 0.2±0.0 |
| **P (WT)** | 100.0±2.6 | 3.2±0.1 | 100.0±3.3 | 3.7±0.4 | 100.0±5.0 | 3.8±0.0 |
| Q | 20.9±1.5 | 0.6±0.0 | 0.1±0.0 | 0.1±0.0 | 9.2±0.0 | 0.3±0.0 |
| R | 18.2±0.1 | 0.2±0.0 | 0.1±0.0 | 0.1±0.0 | 0.6±0.0 | 0.1±0.0 |
| S | 23.6±0.3 | 0.5±0.0 | 0.3±0.0 | 0.1±0.0 | 23.0±0.2 | 0.5±0.1 |
| T | 13±2.3 | 0.2±0.0 | 0.1±0.0 | 0.1±0.0 | 110.1±0.3 | 2.9±0.1 |
| V | 0.1±0.0 | 0.1±0.0 | 0.1±0.0 | 0.1±0.0 | 108.7±7.3 | 2.0±0.3 |
| W | 0.1±0.0 | 0.1±0.0 | 0.1±0.0 | 0.1±0.0 | 4.4±0.0 | 0.1±0.0 |
| Y | 0.7±0.0 | 0.1±0.0 | 0.1±0.0 | 0.1±0.0 | 14.4±1.1 | 0.2±0.0 |

**Table S3. Information on the H (NifH/VnfH/AnfH) proteins used in this study.** A total of 12 NifH, two VnfH, and two AnfH proteins were selected from 12 representative nitrogen-fixing bacteria.

| *H* genes from  diverse origins | Bacterial species | Taxonomy  (phylum) | Protein ID |
| --- | --- | --- | --- |
| *KonifH* | *Klebsiella oxytoca* | Proteobacteria (γ) | WP_004122384.1 |
| *AvnifH* | *Azotobacter vinelandii* | Proteobacteria (γ) | WP_012698831.1 |
| *PsnifH* | *Pseudomonas stutzeri* | Proteobacteria (γ) | WP_003298004.1 |
| *HtnifH* | *Hydrogenobacter thermophilus* | Aquificota | WP_012963773.1 |
| *RcnifH* | *Rhodobacter capsulatus* | Proteobacteria (α) | WP_013066316.1 |
| *AsnifH1* | *Anabaena sp. UTEX B 2576* | Cyanobacteriota | WP_010995626.1 |
| *AsnifH2* | *Anabaena sp. UTEX B 2576* | Cyanobacteriota | WP_010995048.1 |
| *SmnifH* | *Sinorhizobium meliloti* | Proteobacteria (α) | WP_003532770.1 |
| *BsnifH* | *Bacillus sp. 03113* | Bacillota | WP_141432871.1 |
| *PgnifH* | *Paenibacillus graminis* | Bacillota | WP_238326737.1 |
| *PpnifH* | *Paenibacillus peoriae* | Bacillota | WP_023987270.1 |
| *MinifH* | *Methanocaldococcus infernus* | Euryarchaeota | WP_013099459.1 |
| *AvvnfH* | *Azotobacter vinelandii* | Proteobacteria (γ) | WP_012698955.1 |
| *PdvnfH* | *Paenibacillus durus* | Bacillota) | WP_025694161.1 |
| *AvanfH* | *Azotobacter vinelandii* | Proteobacteria (γ) | WP_012703362.1 |
| *RcanfH* | *Rhodobacter capsulatus* | Proteobacteria (α) | WP_013066329.1 |

**Table S4. Absolute acetylene reduction activity of nitrogenase systems carrying different *H* (*nifH*/*vnfH*/*anfH*) genes.** *nifM^+^* and *nifM^−^*, activity obtained in the presence or absence of *nifM*, and error bars represent the mean ± SD of at least three biological replicates.

| *nifH* gene from diver origins | Nitrogenase Activities, ARA  (nM C_2_H_4_/h/OD_600_) | |
| --- | --- | --- |
|  | *nifM^+^* | *nifM^−^* |
| *KonifH* | 185.48±5.88 | 6.35±0.55 |
| *AvnifH* | 110.07±8.90 | 1.34±0.22 |
| *PsnifH* | 136.29±4.17 | 6.38±0.45 |
| *HtnifH* | 13.69±2.68 | 3.97±0.47 |
| *RcnifH* | 47.63±2.58 | 55.79±5.3 |
| *AsnifH1* | 41.36±1.01 | 53.68±2.69 |
| *AsnifH2* | 41.93±1.97 | 58.7±3.62 |
| *SmnifH* | 0.98±0.05 | 1.01±0.15 |
| *BsnifH* | 193.66±19.21 | 165.68±17.75 |
| *PgnifH* | 109.26±9.53 | 95.34±1.08 |
| *PpnifH* | 28.04±1.17 | 25.8±0.23 |
| *MinifH* | 0.21±0.02 | 0.19±0.03 |
| *AvvnfH* | 47.60±4.62 | 0.22±0.03 |
| *PdvnfH* | 39.23±0.83 | 49.12±0.87 |
| *AvanfH* | 78.71±4.33 | 72.00±8.86 |
| *RcanfH* | 32.91±1.69 | 43.35±7.01 |

**Table S5. Absolute acetylene reduction activity of nitrogenase systems carrying chimeric *nifH* genes.** Fragments from KoNifH are shown in blue, and fragments from MiNifH are shown in green. *nifM^+^*/*nifM^−^*, activity obtained in the presence or absence of *nifM*, and error bars represent the mean ± SD of at least three biological replicates.

**Table S6. *nifH*/*vnfH*/*anfH* gene sequences used in this study.** Sequences coding for the N-terminal His-tag are highlighted in blue.

| *nifH/vnfH/anfH*gene | Coding sequence |
| --- | --- |
| *KonifH* | ATGGGCAGCAGCCATCATCATCATCATCACAGCAGCGGAATGACCATGCGTCAATGCGCTATTTACGGTAAAGGCGGTATCGGTAAATCCACCACCACGCAGAACCTCGTCGCCGCGCTGGCGGAGATGGGTAAGAAAGTGATGATCGTCGGCTGCGATCCGAAGGCGGACTCCACCCGTCTGATTCTGCACGCCAAAGCACAGAACACCATTATGGAGATGGCCGCGGAAGTCGGCTCGGTCGAGGACCTCGAACTCGAAGACGTGCTGCAAATTGGCTACGGCGATGTGCGCTGCGCGGAATCCGGCGGCCCGGAGCCAGGCGTCGGCTGCGCGGGACGCGGCGTGATCACGGCGATCAACTTTCTTGAAGAAGAAGGCGCCTACGAGGACGATCTCGATTTCGTGTTCTATGACGTGCTCGGCGACGTGGTCTGCGGCGGCTTCGCCATGCCGATCCGCGAAAACAAAGCCCAGGAGATCTACATCGTCTGCTCCGGCGAAATGATGGCGATGTACGCGGCCAACAATATCTCCAAAGGGATCGTTAAATACGCCAAATCCGGCAAGGTGCGCCTCGGCGGCCTGATCTGTAACTCACGTCAGACCGACCGTGAAGACGAACTGATTATTGCCCTGGCGGAAAAGCTCGGTACCCAGATGATCCACTTTGTGCCCCGCGACAACATCGTGCAGCGCGCGGAGATCCGCCGCATGACGGTTATCGAGTACGACCCCGCCTGTAAACAGGCCAACGAATACCGCACCCTGGCGCAGAAGATCGTCAACAACACCATGAAAGTGGTGCCGACGCCCTGCACCATGGATGAGCTGGAATCGCTGCTGATGGAGTTCGGCATCATGGAAGAGGAAGACACCAGCATCATTGGCAAAACCGCCGCCGAAGAAAACGCGGCCTAA |
| *AvnifH* | ATGGGCAGCAGCCATCATCATCATCATCACAGCAGCGGAATGGCTATGCGTCAATGCGCCATCTACGGCAAAGGTGGTATCGGTAAGTCCACCACTACTCAGAACCTGGTGGCAGCCCTGGCTGAGATGGGCAAGAAGGTCATGATCGTTGGTTGTGACCCGAAAGCTGACTCCACCCGCCTGATCCTGCACTCCAAGGCCCAGAACACCATCATGGAAATGGCTGCCGAAGCCGGTACCGTGGAAGATCTGGAGCTGGAGGACGTGCTGAAGGCTGGCTACGGCGGCGTCAAGTGCGTTGAGTCCGGTGGTCCGGAGCCGGGCGTTGGCTGCGCCGGCCGTGGTGTTATCACCGCCATCAACTTCCTGGAAGAGGAAGGCGCCTACGAAGATGATCTGGACTTCGTATTCTACGACGTGCTGGGCGACGTGGTGTGTGGCGGCTTCGCCATGCCGATCCGCGAGAACAAGGCCCAGGAAATCTACATCGTCTGCTCCGGTGAGATGATGGCCATGTACGCCGCCAACAACATCTCCAAGGGCATCGTGAAGTATGCCAACTCCGGCAGCGTGCGTCTGGGCGGCCTGATCTGCAACAGCCGTAACACTGACCGCGAAGATGAGCTGATCATCGCTCTGGCCAACAAGCTGGGCACCCAGATGATCCACTTCGTGCCGCGTGACAACGTCGTGCAGCGCGCCGAAATCCGCCGCATGACCGTGATCGAATACGATCCGAAAGCCAAGCAAGCCGACGAATACCGCGCTCTGGCCCGCAAGGTCGTCGACAACAAACTGCTGGTCATCCCGAACCCGATCACCATGGACGAGCTCGAAGAGCTGCTGATGGAATTCGGCATCATGGAAGTCGAGGACGAATCCATCGTCGGAAAAACCGCCGAAGAAGTCTAA |
| *PsnifH* | ATGGGCAGCAGCCATCATCATCATCATCACAGCAGCGGAATGGCAATGCGTCAATGCGCTATTTACGGGAAGGGTGGAATCGGCAAATCCACCACGACCCAGAACCTCGTGGCGGCCCTGGCCGAACTCGGCAAGAAGGTCATGATCGTCGGCTGCGACCCCAAGGCCGACTCCACTCGCCTGATCCTGCACTCCAAGGCGCAGAACACCATCATGGAAATGGCCGCCGAGGCCGGTACCGTGGAAGACCTGGAACTCGAGGACGTGCTCAAGACCGGCTACGGCGACATCAAGTGCGTCGAGTCGGGCGGTCCGGAGCCGGGCGTGGGCTGCGCCGGTCGCGGCGTGATCACCGCGATCAACTTCCTCGAAGAGGAAGGCGCCTACGAGGATGACCTGGACTTCGTCTTCTACGACGTGCTCGGCGACGTGGTCTGTGGCGGCTTCGCCATGCCCATCCGCGAGAACAAGGCCCAGGAGATCTACGTGGTCTGCTCCGGCGAGATGATGGCGATGTATGCCGCCAACAACATCTGCAAGGGCATCGTGAAGTACGCCAACTCCGGCAGCGTGCGGCTCGGCGGGCTGATCTGCAACAGCCGCAACACCGACCGCGAGGACGAGCTGATCATGGCCCTGGCCGACAAGCTGGGCTCGCAGATGATCCACTTCGTCCCGCGCGACAACGTCGTGCAGCGCGCCGAAATCCGCCGCATGACCGTCATCGAGTACGACCCCGCCGCCAAGCAGGCCGACGAATACCGGACCCTGGCGAAGAAGATCGTCGAGAACAAGAAACTGGTCATCCCCACCCCGATCAGCATGGACGAGCTGGAAGCCTTGCTTATGGAGTTCGGGATCATGGACGAGGAAGACATGACCATCGTCGGCAAGACCGCCGCCGAGGAAGTCGTTGCCTAA |
| *HtnifH* | ATGGGCAGCAGCCATCATCATCATCATCACAGCAGCGGAATGAGACAAATTGCCATTTACGGTAAGGGTGGTATCGGTAAAAGCACCACCACACAGAATACCGTTGCTGCTTTGGCCGAAGCCGGTAGAAAGTGTTTCATTGTCGGTTGTGATCCGAAAGCTGACTCTACCCGTCTGATTTTGCACGTTAAAGCCCAGAGCACCGTTATGCATCTGGCAGCAGAGCGTGGTGCCGTTGAAGATCTGGATCTGGATGAAGTTATGTTGGTTGGTTTTGGTGGTATCAAATGCGTGGAATCCGGTGGTCCAGAACCAGGTGTTGGCTGTGCTGGTCGTGGTGTTATCACTGCTATCAACTTTCTGGAAGAAAACGGTGCTTTCGATGATGACTTGGATTATGTTTTCTACGACGTCCTTGGTGATGTTGTTTGTGGTGGTTTTGCAATGCCAATTCGTGAAGGTAAGGCACAGGAAATCTATATTGTTACCTCTGGTGAAATGATGGCTATGTACGCTGCAAATAATATATCCAAGGGTATTCTGAAATATGCACATAGCGGCGGTGTCAGATTAGGTGGTCTGATCTGTAATAGCCGTAATGTTGACAACGAAAGAGAATTGATTGAAGCACTGGCAGAAAAGCTGGGTACTCAAATGATTCACTTTTTGCCAAGAAACAACATCGTCCAAGAAGCTGAACTGCGTCGTATGACCGTTATTGAATACGCACCGGATCATCCGATGGCCGATGAATATCGTACCCTGGCAAAAAAAATCGAAGAAAACAGAAAACTGAGCATTCCGACCCCATTGACCATGGACGAATTGGAACAATTGTTGGTTGAATATGGTATTATGAAGCCTGAAGAAGTCGCATAA |
| *RcnifH* | ATGGGCAGCAGCCATCATCATCATCATCACAGCAGCGGAATGGGCAAACTCCGTCAGATCGCCTTCTACGGCAAAGGTGGTATCGGCAAGTCGACCACCTCGCAGAACACCCTCGCCGCGCTGGTCGAGATGGGTCAGAAGATCCTCATCGTCGGCTGCGACCCCAAGGCTGACAGCACCCGTCTGATCCTGAACACCAAGCTGCAGGACACCGTGCTGCACCTGGCCGCCGAGGCCGGTTCGGTCGAAGATCTGGAAGTCGAAGACGTCGTGAAAATCGGCTACAAGGGCATCAAATGCACCGAAGCCGGCGGTCCGGAGCCGGGGGTTGGCTGCGCCGGCCGTGGCGTCATCACCGCGATCAACTTCCTTGAAGAAAACGGCGCCTATGACGATGTGGACTATGTGTCCTATGACGTTCTGGGCGACGTGGTCTGCGGCGGCTTCGCCATGCCGATCCGTGAAAACAAGGCGCAGGAAATCTACATCGTCATGTCGGGCGAGATGATGGCGCTTTACGCCGCCAACAACATCGCCAAGGGCATCCTGAAATATGCGAACTCGGGCGGCGTGCGTCTGGGCGGGCTGATCTGCAACGAACGCAAGACCGACCGCGAGCTGGAACTGGCCGAAGCGCTGGCCGCCAAGCTGGGCTGCAAGATGATCCACTTCGTGCCGCGCAACAACGTCGTGCAACATGCCGAACTGCGCCGCGAAACCGTGATCCAATACGATCCGACCTGCAGCCAGGCGCAGGAATACCGCGAACTGGCCCGCAAGATCCACGAGAACTCGGGCAAGGGCGTCATCCCGACCCCGATCACGATGGAAGAGCTGGAAGAGATGCTGATGGATTTCGGCATCATGCAATCGGAAGAAGATCGCGAAAAGCAGATCGCCGAGATGGAAGCCGCGATGAAGGCCTAA |
| *AsnifH1* | ATGGGCAGCAGCCATCATCATCATCATCACAGCAGCGGAATGACTGACGAAAACATTAGACAGATAGCTTTCTACGGTAAAGGCGGTATCGGTAAATCTACCACCTCCCAAAACACCCTTGCAGCTATGGCAGAAATGGGTCAACGCATCATGATTGTAGGTTGCGACCCTAAAGCTGACTCCACCCGTCTGATGCTTCACTCCAAAGCTCAAACCACCGTACTACACTTAGCTGCTGAACGCGGTGCAGTAGAAGACTTAGAACTCCACGAAGTAATGTTGACCGGTTTCCGTGGCGTTAAGTGCGTAGAATCTGGTGGTCCAGAACCCGGTGTAGGTTGCGCCGGTCGTGGTATCATCACCGCCATTAACTTCTTAGAAGAAAACGGCGCTTACCAAGACCTAGACTTCGTATCCTACGACGTATTGGGTGACGTTGTATGTGGTGGTTTCGCTATGCCTATCCGTGAAGGTAAAGCACAAGAAATCTACATCGTTACCTCTGGTGAAATGATGGCGATGTATGCTGCTAACAACATCGCTCGCGGTATTTTGAAATATGCTCACTCCGGTGGTGTACGTTTAGGTGGTTTGATCTGTAACAGCCGTAAGGTTGACCGTGAAGACGAGTTAATCATGAACTTGGCTGAACGTTTGAACACCCAAATGATTCACTTCGTACCTCGTGACAACATCGTTCAACACGCAGAATTGCGCCGTATGACCGTTAACGAGTACGCACCAGACAGCAACCAAGGTCAAGAGTACCGCGCATTAGCTAAGAAGATCATCAACAACGACAAGCTCACCATTCCTACACCAATGGAAATGGATGAACTAGAAGCTCTGTTGATCGAATACGGTCTATTAGACGACGACACCAAGCACTCTGAAATCATCGGTAAGCCCGCAGAAGCTACCAAATAA |
| *AsnifH2* | ATGGGCAGCAGCCATCATCATCATCATCACAGCAGCGGAATGAGTATCGACAAGAAAATTAGACAAATTGCTTTCTACGGTAAAGGCGGTATTGGTAAGTCTACCACTTCTCAAAACACCTTGGCAGCTATGGCAGAAATGGGTCAACGCATCCTTATCGTAGGTTGCGACCCTAAAGCTGACTCCACCCGTTTGATGCTGCACTCCAAAGCTCAAACCACCGTTCTTCACTTGGCTGCTGAACGTGGTGCAGTAGAAGACCTCGAACTCGAAGAAGTAATGTTGACCGGCTTCCGTGGCGTTAAGTGCGTGGAATCTGGTGGTCCAGAACCCGGTGTAGGTTGCGCTGGTCGTGGTATTATCACCGCCATCAACTTCTTAGAAGAAAACGGCGCTTACCAAGACGTTGATTTCGTATCTTATGACGTATTAGGCGACGTTGTATGCGGCGGTTTCGCAATGCCTATCCGCGAAAATAAAGCGCAAGAAATATACATCGTCACATCAGGTGAAATGATGGCAATGTATGCTGCTAACAACATTGCTCGTGGTATTTTGAAATATGCCCACACTGGTGGTGTACGTTTAGGCGGTTTAATTTGTAACAGCCGTAACGTTGACAGAGAAATCGAACTTATCGAAACTCTGGCAAAACGTTTGAACACCCAAATGATTCACTACGTACCCCGCGACAACATTGTTCAACACGCTGAGTTGCGCCGGATGACAGTTAACGAATACGCACCCGATAGCAACCAAGGTAACGAATACCGGATTTTAGCTAACAAAATCATCAACAACGAAAATCTGAAGATTCCTACCCCAATTGAAATGGAAGAATTAGAAGAGTTGCTGATTGAGTTCGGTATTCTCGAAAGCGAAGAAAATGCTGCAAAAATGATTGCTACAACTTCAGAAAGCAAATCTAAGTAA |
| *SmnifH* | ATGGGCAGCAGCCATCATCATCATCATCACAGCAGCGGAATGGCAGCTCTGCGTCAGATCGCGTTCTACGGTAAGGGGGGTATCGGCAAGTCCACGACCTCCCAAAATACACTCGCCGCGCTTGTCGACCTGGGGCAAAAGATCCTTATTGTCGGCTGCGATCCGAAAGCGGACTCCACGCGCCTCATCCTGAACGCAAAGGCACAGGACACCGTACTGCATCTTGCGGCAACCGAAGGTTCGGTCGAAGACCTCGAGCTCGAGGACGTGCTCAAAGTGGGTTACAGAGGCATCAAGTGCGTGGAGTCCGGTGGCCCAGAGCCGGGCGTCGGCTGCGCCGGACGCGGCGTTATCACCTCGATCAACTTCCTGGAAGAGAACGGCGCTTACAACGATGTCGATTACGTCTCATACGACGTGCTAGGGGACGTAGTATGCGGCGGCTTTGCGATGCCTATTCGCGAAAACAAGGCTCAGGAAATCTACATCGTCATGTCCGGTGAGATGATGGCGCTCTATGCCGCCAACAACATCGCGAAGGGTATCCTGAAGTACGCCCATGCGGGCGGCGTGCGGCTGGGGGGGTTGATTTGCAACGAGCGCCAGACCGATCGGGAGCTCGACCTCGCCGAGGCACTTGCCGCCCGCCTCAATTCCAAGCTCATCCACTTCGTGCCGCGCGACAATATCGTTCAGCACGCAGAGCTCAGAAAGATGACAGTGATCCAATATGCGCCGAACTCTAAGCAAGCCGGGGAATATCGCGCCCTGGCTGAAAAGATCCATGCAAATTCCGGCCGAGGCACCGTCCCTACACCGATCACTATGGAGGAACTGGAGGACATGCTGCTCGACTTTGGAATCATGAAGAGCGACGAGCAGATGCTTGCCGAACTCCACGCCAAGGAAGCCAAGGTAATAGCCCCCCACTAA |
| *BsnifH* | ATGGGCAGCAGCCATCATCATCATCATCACAGCAGCGGAATGAGACAAATCGCTTTCTATGGTAAAGGTGGAATTGGTAAATCAACAACTTCTCAAAATACTTTAGCGCAATTAGCAACAAAATTCGGTCAAAAAATTATGATTGTAGGCTGTGACCCTAAAGCAGATTCAACTCGTTTAATTTTAAATACAAAAGCACAAAGCACCGTATTAGAAAAGGCTGCGGAAATGGGTACGGTTGAGGATTTAGAATTAGAAGATGTTGTAGCAAAAGGTTATGGAGATATTTTATGTGTGGAATCAGGCGGACCTGAGCCAGGAGTAGGCTGTGCTGGACGCGGAATTATCACTTCTATCAATTTCCTAGAAGAGGAAGGTGCCTATGAAGGACTTGACTTTGTCTCTTATGACGTATTAGGGGACGTTGTGTGTGGTGGATTTGCGATGCCAATTCGTGAAAACAAAGCACAGGAAATCTATATCGTTTGTTCAGGTGAAATGATGGCGATGTATGCAGCAAATAATATTTCTCGCGGTATTTTAAAATATGCCAATAGCGGCGGTGTTCGTTTAGGTGGTTTAATCTGTAACAGCCGTAACACTGACCGTGAAGCTGAGCTTATTTCAGAGCTTGCTCGTCGTTTAAATACACAAATGATCCACTATGTACCACGTAATAATATTGTTCAACATGCTGAGTTGCGTAAAATGACCGTTGCTCAATACAGACCAGAAGATGAACAAGCAAAAGAGTATGAATATTTAGCTAAAAAAATATTAGAAAATGAAATGATGACAATTCCAACACCAATTGAAATGGACGAATTAGAATCATTGCTTATGGAATTTGGTGTTATTGAAGATGAAGAAACTGCAATTAAAAAGCTAGAAGAAAAAGAAGCGGCTGGTAACTAA |
| *PgnifH* | ATGGGCAGCAGCCATCATCATCATCATCACAGCAGCGGAATGAGACAAATAGCTTTCTACGGTAAAGGCGGTATCGGTAAATCCACAACTTCCCAGAACACTTTGGCCCAGCTCGCAACCAAATTCAAACAAAGAATTATGATCGTTGGCTGTGACCCGAAGGCAGACTCCACACGCCTGATTCTGAACACGAAAGCACAGAACTCGGTACTGGAGCTGGCAGCCGAACTGGGCTCGGTTGAGGATTTGGAACTCGAAGATGTGCTTCAGACCGGCTTTGGCGACATCATCAACGTAGAGTGCGGCGGGCCTGAACCGGGTGTAGGCTGTGCAGGGCGCGGTATCATTACTGCCATCAACTTCCTGGAGCAGGAAGGGGCTTATCAGGATCTGGACTTCGTATCCTATGACGTACTGGGTGACGTTGTATGCGGCGGGTTCGCAATGCCTATCCGTGAAGGCAAGGCACAAGAGATTTATATTGTCTGTTCCGGAGAAATGATGGCCATGTATGCAGCCAACAACATTGCACGCGGTATCCTGAAATATGCTACCAGCGGCGGCGTAAGACTGGGCGGACTGATCTGCAACAGCCGTAACACCGACCGTGAAGATGAGCTGATCATGGAGCTGGCCCGCCGTCTGAACACGCAAATGATCCACTTCGTCCCCCGGGATAATATCGTTCAACATGCCGAGCTGCGCAGAATGACGGTCGCCCAATATAATCCTGCCCATCAACAAGCCAAAGAATATGAAATCCTGGCTGAAAAAATCCTCAACAACAAAATGCTGACTATCCCTACTCCGATCTCCATGGAAGAGCTGGAAGAGCTGCTGATGGAATTCGGCATCATCGAAGATGAAGAAGCAGCGATCAAGAAGCTGAAGGCTTCCGGCCAATAA |
| *PpnifH* | ATGGGCAGCAGCCATCATCATCATCATCACAGCAGCGGAATGAGACAAATTGCGTTTTACGGTAAGGGCGGTATCGGCAAATCGACAACCTCGCAGAATACACTGGCTCAACTCGCGACCAAATTCAAACAAAAAATTATGATCGTAGGCTGTGATCCCAAGGCAGACTCCACCCGTCTTATTCTGAATACGAAGGCCCAACAGACTGTGCTGCATCTGGCAGCTGAAAGGGGTACGGTGGAGGACTTGGAACTGGAGGATGTTGTCCAGAAGGGCTTCGGTGATATTCTGAACGTGGAATGCGGCGGGCCAGAGCCCGGTGTCGGCTGTGCAGGACGCGGTATCATCACAGCCATTAATTTTCTGGAGGAAGAGGGGGCCTACGAAGGGCTGGATTTCGTATCCTACGATGTACTGGGGGACGTCGTGTGCGGGGGGTTCGCCATGCCGATCCGGGAGAAGAAGGCGCAGGAAATCTACATTGTATGCTCAGGCGAGATGATGGCTATGTACGCTGCCAACAATATTGCGCGCGGGATCTTGAAGTATGCCAACAGCGGCGGGGTGCGTTTGGGCGGCTTAATCTGCAACAGCCGGAATACGGACCTGGAAGCGGAATTGATCACAGAGCTTGCAAGAAGATTGAACACGCAGATGATCCACTTTTTGCCGCGTGACAATGTTGTGCAGCACGCTGAGCTGCGCCGTATGACCGTTACCCAATATAACCCGGAACATAAGCAGGCTGCGGAGTATGAAGAGCTGGCAGGTAAGATTTTGAATAATGACATGCTAACGGTTCCCACGCCCATTTCCATGGAAGATCTGGAGGATCTATTGATGGAATTCGGCATTATTGAGGATGAAGAAACCGCAATTAACAAAGCTGAGGCGTCCGGGCAGTAA |
| *MinifH* | ATGGGCAGCAGCCATCATCATCATCATCACAGCAGCGGAATGTCATTTGATGAAATAGCTCCCAATGCAAAGAAAGTTGCGATCTACGGCAAGGGCGGTATTGGTAAGTCCACCACGACGCAAAACACCGCTGCCGCGTTGGCGTATTATTACAAGCTGAAGGGTATGATTCACGGTTGTGACCCTAAAGCGGATAGCACCCGTATGATCCTGCATGGTAAGCCGCAAGAAACCGTTATGGACGTGCTGCGTGAGGAAGGCGAAGAGGGCGTCACCCTGGAGAAGGTGCGCAAAGTGGGTTTCTGCGGTATCTACTGCGTTGAGAGCGGCGGCCCAGAACCGGGTGTGGGCTGCGCTGGTCGTGGTGTGATTACCGCGGTGAACCTGATGAAAGAACTGGGTGGCTATCCGGACGACCTGGATTTCCTCTTTTTTGATGTTCTGGGTGACGTCGTGTGCGGTGGTTTTGCCATGCCGCTGCGTGACGGTCTGGCGAAAGAGATCTACATCGTGTCGTCCGGTGAGATGATGGCACTGTACGCGGCTAATAACATCGCGAAAGGCATCCTCAAATACGCGGAGCAGAGCGGTGTACGCCTGGGTGGCATTATTTGTAATAGCCGTAATGTTGATGGCGAGCGCGAATTAATGGAAGAGTTCTGCGACAAGCTGGGTACCAAACTTATCCACTTTATTCCGCGTGATAACATTGTTCAGAAAGCTGAATTCAACAAAATGACCGTTGTTGAATTCGCACCGGATCATCCGCAGGCACTGGAGTATAAAAAATTGGGCAAGAAGATCATGGACAACGATGAATTGGTTATTCCGACTCCGTTGTCTATGGACGAACTTGAGAAGTTGGTCGAGAAGTACGGCCTGTATGATAAATAA |
| *AvvnfH* | ATGGGCAGCAGCCATCATCATCATCATCACAGCAGCGGAATGGCATTGCGTCAGTGTGCAATTTACGGCAAGGGTGGCATCGGCAAGTCCACCACCACCCAGAACCTGGTCGCCGCCCTCGCCGAAGCCGGCAAGAAAGTGATGATCGTCGGTTGTGACCCGAAAGCCGACTCCACCCGCCTGATCCTGCACTCCAAGGCCCAGGGCACCGTCATGGAAATGGCCGCGTCCGCCGGCTCGGTCGAAGACCTGGAGCTGGAAGACGTGCTGCAGATCGGCTTCGGCGGCGTCAAGTGCGTCGAATCCGGTGGCCCGGAGCCGGGCGTCGGCTGCGCCGGCCGTGGCGTGATCACCGCGATCAACTTCCTGGAAGAAGAAGGCGCCTACAGCGACGACCTGGACTTCGTGTTCTATGACGTGCTGGGCGACGTGGTATGCGGCGGCTTCGCCATGCCGATCCGCGAGAACAAGGCCCAGGAAATCTACATCGTCTGCTCCGGCGAGATGATGGCCATGTACGCCGCCAACAACATCGCCAAGGGCATCGTGAAATACGCCCACTCCGGCAGCGTGCGTCTGGGCGGCCTGATCTGCAACAGCCGCAAGACCGACCGCGAAGACGAGCTGATCATGGCCCTGGCCGCGAAGATCGGCACCCAGATGATCCACTTCGTGCCGCGCGACAACGTCGTGCAACACGCCGAAATCCGCCGCATGACCGTGATCGAATACGATCCGAAGGCCGGACAGGCCGACGAGTACCGTGCCCTGGCTCGCAAAATCGTCGACAACAAGCTGCTGGTCATCCCGAACCCGGCCTCCATGGAAGAACTCGAAGAGCTGCTGATGGAATTCGGCATCATGGAAGTCGAAGACGAGTCCGTCGTCGGCAAGGCCGCCGCCGAAGGCTAA |
| *PdvnfH* | ATGGGCAGCAGCCATCATCATCATCATCACAGCAGCGGAATGACAAGAAAAATCGCAATTTACGGTAAGGGCGGCATTGGTAAATCCACAACACAACAAAACACGGCTTCGGCAATGGCTTATTTCCACAAAAAACAGGTGTTCATCCATGGTTGCGATCCGAAGGCCGACTCCACCCGGATGATTCTCGGCGGCAAACCGCAAGAAACATTGATGGATACGCTGCGCGAAACCGGTGAAGAATCCATCACGGTCGACAGTGTAGTAAAAACGGGTTATCAAGGTATCCGCTGCGTTGAATCAGGCGGTCCGGAACCGGGTGTTGGTTGTGCGGGACGCGGCGTTATTACCGCAATCAACCTGATGGAAGATCTTGGTGCTTACACTGAGGACCTCGACTTTGTATTCTTTGACGTATTGGGCGACGTTGTATGCGGCGGTTTCGCAATGCCGATTCGCGAAGGCAAAGCAGAAGAGGTTTACATCGTGGCCTCCGGTGAGATGATGGCTATTTACGCAGCTAACAATATTGCACGCGGTATGCTGAAATATGCGGATCAGAGCGGCGTCCGGCTGGGCGGTATTATCTGTAACAGCCGTAACGTTGATAAAGAATTAGAACTTATGGAAGAATTCACTTCCAAGCTTGGCACTCAACTGATTCACTTTGTACCACGCGACAACATCGTTCAAAAGGCGGAATTCAATAAAAAATCGGTTATCGAATTTGACGCTGCAAGTAATCAAGCTAATGAATATTCAGAACTGGCACGTAAAATTATCGAAAACAAAAGCTTCGTAATTCCTACTCCGCTGACCATGGACGAGCTGGAAGATATGGTTACCAAGTACGGTATTGTCGACTAA |
| *AvanfH* | ATGGGCAGCAGCCATCATCATCATCATCACAGCAGCGGAATGACTCGTAAAGTAGCCATTTACGGAAAAGGCGGTATCGGCAAATCCACCACTACCCAGAATACTGCCGCAGCGCTGGCCTATTTCCACGACAAGAAAGTCTTCATCCACGGCTGCGACCCCAAGGCGGACTCCACCCGCCTGATTCTCGGCGGCAAACCTCAGGAAACCCTGATGGACATGCTGCGCGACAAGGGCGCCGAAAAGATCACCAACGACGACGTGATCAAAAAAGGCTTTCTGGACATCCAGTGCGTGGAGTCCGGCGGCCCCGAGCCGGGCGTTGGTTGCGCTGGCCGCGGCGTGATCACCGCCATCGACCTGATGGAAGAAAACGGCGCCTATACCGATGACCTGGATTTCGTGTTCTTCGACGTACTGGGCGACGTCGTGTGCGGCGGTTTCGCCATGCCGATCCGCGACGGCAAGGCCCAGGAAGTCTACATCGTGGCTTCCGGGGAGATGATGGCCATTTATGCGGCCAACAACATCTGCAAGGGCCTGGTGAAATACGCCAAACAAAGTGGCGTGCGCCTGGGCGGCATCATTTGCAACAGCCGTAAGGTGGATGGCGAGCGCGAGTTCCTGGAAGAGTTCACCGCGGCCATCGGTACCAAGATGATCCACTTCGTTCCGCGCGACAATATCGTGCAGAAGGCCGAGTTCAACAAGAAGACCGTGACCGAGTTCGCTCCAGAGGAAAACCAGGCCAAGGAGTACGGCGAGCTGGCCCGCAAGATCATTGAGAACGATGAATTCGTCATTCCCAAACCGCTGACCATGGACCAACTGGAAGACATGGTCGTCAAGTACGGTATTGCCGACTAA |
| *RcanfH* | ATGGGCAGCAGCCATCATCATCATCATCACAGCAGCGGAATGACCCGCAAGATCGCCATTTACGGCAAAGGTGGTATCGGCAAATCGACGACCACCCAGAACACCGCCGCAGCCCTTGCCTTTTTCCACGAAAAGAACGTCTTCATCCACGGCTGTGACCCGAAAGCCGACAGCACCCGGCTGATCCTGGGCGGTCTGCCGCAGCAGACGGTGATGGACACGCTGCGCATCGAGGGCGCCGAGCGCGTCACCGTGGACAAGGTCGTGAAGACCGGCTTCAAGGACATCCGCTGCGTGGAATCGGGCGGGCCGGAGCCGGGCGTGGGCTGCGCCGGTCGCGGCGTCATCACCGCCATCGACCTGATGGAAGAAAACGAAGCCTACAGCGAAGACCTTGATTTCCTGTTCTTCGACGTGCTGGGCGACGTGGTTTGCGGCGGCTTCGCCATGCCCATTCGCGACGGCAAGGCGGAGGAAGTCTATATCGTCGCCTCGGGCGAGATGATGGCGATCTATGCCGCCAACAACATCTGCAAGGGTCTGGCGAAATACGCCCGGCAATCGGGCGTGCGTCTGGGCGGGATCATCTGCAACAGCCGCAATGTCGATGGCGAAAAGGAATTCCTTGAGGAATTCACCAAGGCCATCGGCACCAAGATGATCCACTTCGTGCCGCGCGACAACATCGTGCAAAAGGCCGAGTTCAACAAGCAGACCGTGACCGAATTCCAGCCCGAGGCCAATCAGGCGCAGGAATACCGCGAACTCGGCCGCAAGATCATCGAGAACGAGGATTTCGTGATCCCGAAGCCGCTCGCCATGGATGAGCTGGAAGCCATGGTCGTCAAATACGGCCTGATGGACTAA |

**SI Reference**

1. Varadi M, Bertoni D, Magana P, Paramval U, Pidruchna I, Radhakrishnan M, Tsenkov M, Nair S, Mirdita M, Yeo J, Kovalevskiy O, Tunyasuvunakool K, Laydon A, Žídek A, Tomlinson H, Hariharan D, Abrahamson J, Green T, Jumper J, Birney E, Steinegger M, Hassabis D, Velankar S. 2024. AlphaFold Protein Structure Database in 2024: providing structure coverage for over 214 million protein sequences. Nucleic Acids Research 52:D368-D375.

2. Pierce B, Tong W, Weng Z. 2005. M-ZDOCK: a grid-based approach for Cn symmetric multimer docking. Bioinformatics 21:1472-1478.

3. Landau M, Mayrose I, Rosenberg Y, Glaser F, Martz E, Pupko T, Ben-Tal N. 2005. ConSurf 2005: the projection of evolutionary conservation scores of residues on protein structures. Nucleic Acids Research 33:W299-W302.

4. Robert X, Gouet P. 2014. Deciphering key features in protein structures with the new ENDscript server. Nucleic Acids Research 42:W320-W324.

5. Strop P, Takahara PM, Chiu H-J, Angove HC, Burgess BK, Rees DC. 2001. Crystal Structure of the All-Ferrous [4Fe-4S]0 Form of the Nitrogenase Iron Protein from Azotobacter vinelandii. Biochemistry 40:651-656.

6. Maslać N, Cadoux C, Bolte P, Murken F, Gu W, Milton RD, Wagner T. 2024. Structural comparison of (hyper-)thermophilic nitrogenase reductases from three marine Methanococcales. The FEBS Journal 291:3454-3480.
